# Phase Stability of Iron Nitride Fe_4_N at High Pressure—Pressure-Dependent Evolution of Phase Equilibria in the Fe–N System

**DOI:** 10.3390/ma14143963

**Published:** 2021-07-15

**Authors:** Marius Holger Wetzel, Tina Trixy Rabending, Martin Friák, Monika Všianská, Mojmír Šob, Andreas Leineweber

**Affiliations:** 1 Institute of Materials Science, Technische Universität Bergakademie Freiberg, Gustav-Zeuner-Str. 5, D-09599 Freiberg, Germany; marius.wetzel@iww.tu-freiberg.de; 2 Freiberg High Pressure Research Centre (FHP), Technische Universität Bergakademie Freiberg, Lessingstraße 45, D-09599 Freiberg, Germany; 3 Institute of Inorganic Chemistry, Technische Universität Bergakademie Freiberg, Leipziger Straße 29, D-09599 Freiberg, Germany; tina.rabending@chemie.tu-freiberg.de; 4 Institute of Physics of Materials, Czech Academy of Sciences, v.v.i., Žižkova 22, CZ-616 62 Brno, Czech Republic; friak@ipm.cz (M.F.); monika.vsianska@ceitec.muni.cz (M.V.); mojmir@ipm.cz (M.Š.); 5 Department of Chemistry, Faculty of Science, Masaryk University, Kotlářská 2, CZ-611 37 Brno, Czech Republic

**Keywords:** iron nitride, high pressure, phase stability, phase transitions, phase diagrams, phase equilibria

## Abstract

Although the general instability of the iron nitride γ′-Fe_4_N with respect to other phases at high pressure is well established, the actual type of phase transitions and equilibrium conditions of their occurrence are, as of yet, poorly investigated. In the present study, samples of γ′-Fe_4_N and mixtures of α Fe and γ′-Fe_4_N powders have been heat-treated at temperatures between 250 and 1000 °C and pressures between 2 and 8 GPa in a multi-anvil press, in order to investigate phase equilibria involving the γ′ phase. Samples heat-treated at high-pressure conditions, were quenched, subsequently decompressed, and then analysed ex situ. Microstructure analysis is used to derive implications on the phase transformations during the heat treatments. Further, it is confirmed that the Fe–N phases in the target composition range are quenchable. Thus, phase proportions and chemical composition of the phases, determined from ex situ X-ray diffraction data, allowed conclusions about the phase equilibria at high-pressure conditions. Further, evidence for the low-temperature eutectoid decomposition $\gamma^{\prime }\to \alpha +\varepsilon^{\prime }$ is presented for the first time. From the observed equilibria, a *P*–*T* projection of the univariant equilibria in the Fe-rich portion of the Fe–N system is derived, which features a quadruple point at 5 GPa and 375 °C, above which γ′-Fe_4_N is thermodynamically unstable. The experimental work is supplemented by ab initio calculations in order to discuss the relative phase stability and energy landscape in the Fe–N system, from the ground state to conditions accessible in the multi-anvil experiments. It is concluded that γ′-Fe_4_N, which is unstable with respect to other phases at 0 K (at any pressure), has to be entropically stabilised in order to occur as stable phase in the system. In view of the frequently reported metastable retention of the γ′ phase during room temperature compression experiments, energetic and kinetic aspects of the polymorphic transition $\gamma^{\prime }⇌\varepsilon^{\prime }$ are discussed.

## 1. Introduction

The large body of research devoted to the Fe–N system is mainly motivated by its high technological relevance in the field of surface treatment of iron and steel parts [1]. Although studies covering microstructure evolution and mechanical properties of nitrided surface layers predominate, there has been, and still exists, a growing interest in the fundamental physical properties of Fe-based nitride phases. This can be attributed to the peculiar magnetic properties of the iron nitrides contrasting the magnetism of the allotropes of pure Fe. The Fe–N system offers a unique opportunity to study phase stability of the various allotropes of Fe (α-bcc, γ-fcc, and ε-hcp) being subject to modified stability by the presence of interstitial atoms. In contrast to the Fe–C system, the Fe–N system is ideal for such studies due to the existence of structural similarity between the terminal solid solutions and the interstitial compounds. The structures of Fe–N phases up to the composition of Fe_2_N can be understood to be composed of a fully occupied bcc, fcc, or hcp sublattice of Fe atoms with N on octahedral sites. In the terminal solid solution phases α-Fe(N) with bcc Fe arrangement and γ-Fe(N) with fcc Fe arrangement, the N atoms are distributed randomly over the interstitial sublattice. The same can be expected for a potential ε-Fe(N) solid solution of the hcp high-pressure modification ε-Fe. In contrast to this, the nitride phases γ′-Fe_4_N and ε′-Fe_3_N_1+*x*_ show long-range order of N on the interstitial sublattice [2,3,4,5]. Crystallographic information on the phases relevant for this work is listed in Table 1. Throughout this text, Greek letters with prime symbols, e.g., γ′ and ε′, will be used to refer to nitride phases with a potentially long-range ordered arrangement of N atoms, and plain Greek symbols to refer to terminal solid solution phases α, γ, and ε. In the case of the ε′ nitride phase, this is not common practice [6], but it helps to establish consistent labelling of the relevant phases.

**Figure 1:**
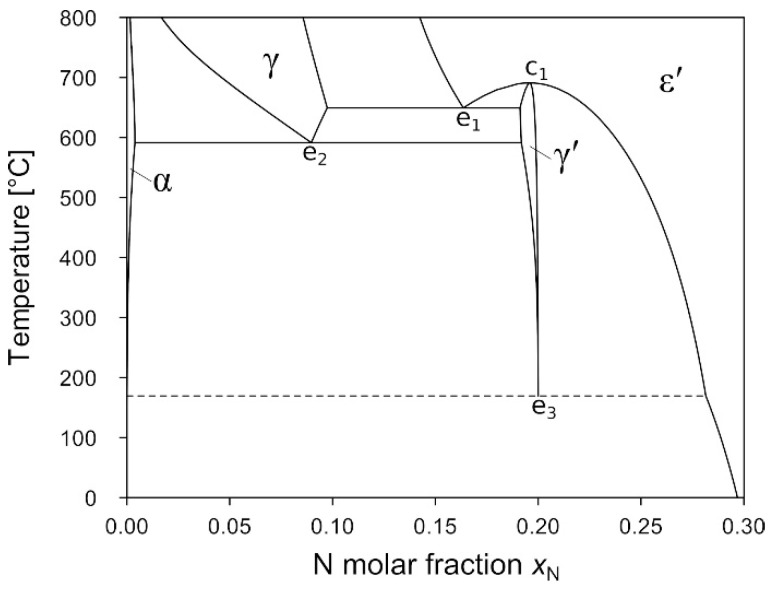
Phase diagram of the Fe-rich part of the Fe–N system calculated for ambient pressure conditions using the Thermo-Calc software [11] employing the database provided by Göhring et al. [7]. See text for an explanation of designations c_1_ and e_1…3_.

At atmospheric pressure, the formation of N-rich phases, such as the nitride phases γ′ and ε′, is typically accomplished by gaseous nitriding (using a NH_3_ + H_2_ mixture), salt bath nitriding, or plasma nitriding processes. All processes impose a high N activity on Fe, which is multiple orders of magnitude higher than the N activity provided by N_2_ gas at atmospheric pressure. This in turn means that Fe nitrides are generally metastable with respect to decomposition into Fe and N_2_ at the nitriding temperatures. Currently accepted thermodynamic databases [7,8] represent the metastable phase equilibria in the Fe–N system with a topology similar to that shown in Figure 1. It is generally accepted that the γ′ phase transforms to $\varepsilon^{\prime }$ by a congruent reaction $\varepsilon^{\prime }⇌\gamma^{\prime }$ (c_1_) and that there are at least two more eutectoid reactions, $\varepsilon^{\prime }⇌\gamma +\gamma^{\prime }$ (e_1_) and $\gamma ⇌\alpha +\gamma^{\prime }$ (e_2_). In contrast, information on the low-temperature eutectoid decomposition $\gamma^{\prime }⇌\alpha +\varepsilon^{\prime }$ (e_3_) is more vague, due to its expectedly low equilibrium temperature [9,10]. Throughout this text the notation α⇌β will be used to refer to a univariant equilibrium reaction of a phase α which is stable at high temperatures to a phase β which is stable at low temperatures. A reaction equation β→α indicates a specific direction of the reaction, e.g., in a pressure-induced phase transformation of the phase β being stable at low pressure to the phase α stable at high pressure.

Due to the high relevance of Fe and Fe-based alloys to the field of materials science, the high-pressure allotropic transformations of Fe have been subject of numerous experiment-based [12,13,14] and theory-based studies [15,16,17]. The first high-pressure research on iron nitrides has been triggered by their peculiar volume-dependent magnetic properties. This can best be exemplified for the γ-Fe(N) and γ′-Fe_4_N phases. Incorporation of N atoms in the octahedral sites of γ-Fe leads to a volume increase and the stabilisation of the γ solid solution to temperatures much lower than the allotropic transformation γ⇌α. The ferromagnetic γ′ phase has a molar volume which is by 20% higher than that of pure γ-Fe, and is about 4.3% higher than a hypothetical paramagnetic γ-Fe(N) of similar composition. This renders γ′-Fe_4_N the ideal prototype to study the volume dependence of magnetic properties of both binary and ternary transition metal nitrides [18,19,20,21].

Further, a series of works with background in the field of geosciences cover the stability of Fe nitrides at high-pressure conditions [22,23,24,25]. It has been shown implicitly by Minobe et al. [25] that the γ′ phase can be retained upon compression to 99 GPa. This metastable retention upon RT compression has occasionally been interpreted to indicate that the γ′ phase is thermodynamically stable at RT and high-pressure conditions [24,26]. In contrast, it has been shown in several other works that a phase transition $\gamma^{\prime }\to \varepsilon^{\prime }$ can be induced upon annealing at *T* > 900 °C at a pressure in the GPa range [23,27], and that moderate heating at 400 °C at 20–30 GPa could induce a transition $\gamma^{\prime }\to \gamma +\varepsilon^{\prime }$ [23]. It should be noted that the potential existence of a $\gamma^{\prime }\to \varepsilon^{\prime }$ transition at high pressure already becomes evident from Figure 1 as a congruent reaction $\varepsilon^{\prime }⇌\gamma^{\prime }$ or, in general, an extension of the ε′ region to N contents below Fe_4_N is already present in the system at atmospheric pressure.

Despite the numerous works on the compression behaviour of Fe–N phases, including the study of phase transitions [25,27,28] and high-pressure synthesis of novel Fe nitrides [29,30,31] (mostly of higher N contents than relevant for the present work), only a small number of works have actually tried to derive any data on the phase equilibria present in the system at elevated pressure. In our previous work [32] phase equilibrium data of the Fe–N system at 13 GPa have been determined. Further, it was found that the iron nitride γ′-Fe_4_N decomposes into low-N γ solid solution and high-N ε′ nitride if heat-treated between 400–600 °C at 13 GPa, whereas at *T* ≥ 700 °C a transformation into ε′-Fe_3_N_0.75_ takes place. By means of Clapeyron-slope estimations it has been shown that, with increasing pressure, the phase region of the γ′ nitride is continuously constricted from both low- and high-temperature sides, and could likely disappear at *P* < 8 GPa [32].

Somewhat before our experimental study at 13 GPa, Breton et al. [24] published an alternative *P–T* projection of phase equilibria in the Fe–N system covering the pressure range of 15–60 GPa and temperature range of 300–1600 K. The derived *P*–*T* projection shows an invariant point involving γ′-Fe_4_N at 41 GPa and 1000 K and implies thermodynamic stability (or re-appearance) of the γ′-Fe_4_N up to 56 GPa at 300 K, apparently contradicting experimental results at high pressure [23,32] and currently accepted thermodynamic models of the system at atmospheric pressure [7,8].

Controversy concerning the phase equilibria in the Fe–N system at elevated pressure exists mainly as, presently, no phase equilibrium data in the pressure range relevant for the pressure-induced disappearance of the γ′ phase exists. In our own and other previous works experiments have been conducted at pressures [23,32] or temperatures [27] far higher than the conditions anticipated for thermodynamic stability of the γ′ phase [32].

In the present study, a more detailed experimental investigation of the pressure induced disappearance of the γ′-Fe_4_N has been conducted in the relevant pressure and temperature range, i.e., at *P* ≤ 8 GPa and *T* < 700 °C. The obtained data are cast into phase diagrams used to derive a more definite, experiment-based *P*–*T* projection of the univariant equilibria in the Fe–N system up to a composition of Fe_3_N. Employing ab initio calculations and literature data, thermodynamic aspects of the stability of the Fe–N phases with respect to pressure and temperature are discussed, with special emphasis on the γ′-Fe_4_N phase.

## 2. Materials and Methods

### 2.1. DFT Calculations

**Figure 2:**
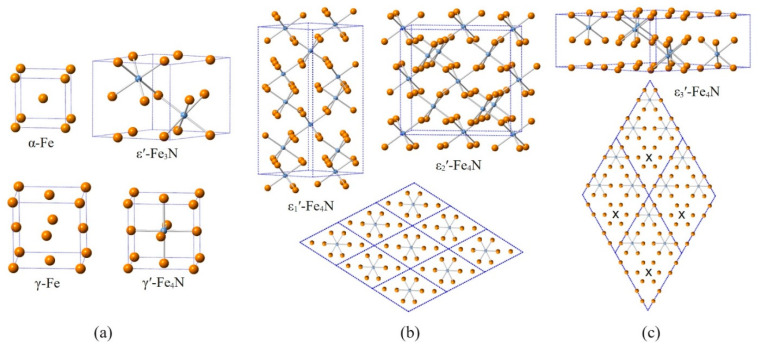
Illustration of the crystal structures relevant for the present work (see also Table 1 and Table 2). “Bonds” highlight the nearest neighbour N–Fe distances. The unit cells are outlined by blue dotted lines. (**a**) Crystal structures of the phases given in Table 1 ignoring the disordered N sites in α/γ-Fe(N) and reflecting the ideal N ordering in γ′-Fe_4_N/ε′-Fe_3_N (see also Table 2). (**b**,**c**) Different $\varepsilon_{1}^{\prime }$-, $\varepsilon_{2}^{\prime }$-, and $\varepsilon_{3}^{\prime }$-Fe_4_N model structures as listed in Table 2, showing the unit cell contents augmented by further Fe atoms completing the octahedral environments of the depicted N atoms. The hexagonal *c* axes of the underlying hcp structures are oriented vertically as also in the case of ε′-Fe_3_N. At the bottom of (**b**,**c**), single layers of octahedral sites perpendicular to the *c* axis of the underlying hcp structure are shown for (**b**) $\varepsilon_{1}^{\prime }$-Fe_4_N (similar for $\varepsilon_{2}^{\prime }$-Fe_4_N) and (**c**) $\varepsilon_{3}^{\prime }$-Fe_4_N, depicting the different types of distributions of the N atoms in these model structures. The “x” in (**c**) marks the empty octahedral sites, which are additionally occupied in ε′-Fe_3_N showing the similarity between both structures, while the N ordering in ε_1_′- and ε_2_′-Fe_4_N resembles that in γ′-Fe_4_N. Within a (001)_hcp_ layer the N ordering implies a doubling of the two-dimensional periodicity, as it is the case for the (111)_fcc_ plane in the case of γ′-Fe_4_N. The $\varepsilon_{2}^{\prime }$-Fe_4_N model structure seems to correspond to a hcp base Fe_4_C structure predicted recently to be stable in the Fe-C system [40].

The quantum-mechanical calculations were performed using the Vienna Ab initio Simulation Package (VASP) [33,34] that implements the density functional theory (DFT) [35,36]. Projector augmented wave (PAW) pseudopotentials [37,38] (Fe_pv: 3pd7s1 and N: s2p3) were employed in the calculations. The plane wave cut-off energy amounted to 500 eV and the product of the number of atoms and the number of k-points in the reciprocal space was between 12,000 and 15,000. The exchange and correlation energy were treated within the generalised gradient approximation (GGA) as parametrised by Perdew, Burke, and Ernzerhof (PBE) [39]. Volumes and shapes of the supercells were relaxed in the calculations. Relaxations were terminated when atomic forces smaller than 0.0005 eV/Å were obtained. The resulting structures are shown in Figure 2 and respective crystallographic information is given in Table 2.

### 2.2. Starting Materials

Fe powder (Alfa Aesar, 99.9+% metals basis, particle size < 10 µm) has been gas nitrided for 150 h at 510 °C in a flowing NH_3_/H_2_ gas mixture in a technical nitriding furnace (Härterei Carl Gommann GmbH, Remscheid, Germany) to prepare single phase γ′-Fe_4_N powder. To prepare samples with compositions of approximately 15 at%, some of the γ′ powder was mixed with the same pure α-Fe powder as was used for the nitriding process. Pure γ′ powder, as well as the α + γ′ powder mixture, were pre-compressed with a hydraulic press into dense pellets of 3.0 mm diameter and 2.2–2.3 mm height. The pressed powder pellets were sealed into CsCl capsules to avoid N-loss and oxygen contamination of the samples during the high-pressure heat treatments. The capsules were die-forged from water-free, annealed CsCl (Merck KGaA, 99.5% purity) to the final dimensions of 4.2 mm outer diameter, 3.3–3.4 mm height (depending on the pressed powder pellet) and 0.5–0.6 mm wall thickness. The entire sample storage, preparation, sealing, and mounting into the octahedral pressure medium (see Section 2.3) was carried out in a glovebox operated with an N_2_ gas atmosphere.

### 2.3. Multi-Anvil Experiments

Two different assembly geometries, namely 18/12 and 14/8 were employed in the experiments (see Supplementary Materials for sketches). The first involves octahedral pressure media with an edge length of 18.5–18.7 mm and WC-Co anvils with 12 mm edge length truncations, and the second octahedra with 14.5–14.7 mm edge length and 8 mm truncations, respectively. Assemblies of type 18/12 were used in experiments at 2 GPa, whereas assemblies of type 14/8 were used for experiments at higher pressures.

Both the octahedral pressure media and the plugs above the sample capsule were manufactured from monoclinic ZrO_2_ (Saint-Gobain; Le Pontet, France). ZrO_2_, which is commonly used as thermal insulation sleeve in multi-anvil experiments allows for better thermal insulation than the commonly used MgO at the expense of a potentially reduced maximum achievable pressure [41].

The central hole in the octahedra was lined with a Kanthal A1 foil with 65 µm thickness, which served as resistive heater. The filled capsules placed in the centre position of the lined bore hole. Type C thermocouples (W5%Re/W26%Re, ConceptAlloys, Inc., Whitmore Lake, MI, USA) were used to record the temperature during annealing. The thermocouple junction was held in position by an Al_2_O_3_ capillary tube with 1.2 mm diameter (Friatec GmbH, Mannheim, Germany) which was positioned in a central hole in the ZrO_2_ plug and in direct contact to the CsCl capsule. Special care was taken to ensure identical thermocouple positions along the furnace in any of the experiments. Coils of bare copper wire, 6–7 mm in length, were inserted ≈1 mm deep into the octahedron to allow for mechanical protection along the 5 mm wide and 3.0–3.1 mm thick Micanite gaskets.

All experiments were conducted in a uniaxial hydraulic press (10 MN maximum ram force) equipped with a Walker-type [42] multi-anvil module (both Voggenreiter Sondermaschinen GmbH, Mainleus, Germany) to generate quasi-hydrostatic pressure conditions.

Table 3 summarises the conditions of each multi-anvil experiment. The sample pressure has been determined by comparison with calibration curves determined in additional calibration experiments using the phase transitions of Bi [43,44] as well as ZrO_2_ [45] (see Supplementary Materials and Reference [46] for more information on the calibration procedure). Heat treatments were started after the samples have been compressed to the target pressure. The given temperatures are those directly measured by the thermocouple. No corrections for the potential effects of pressure [47] or protective copper coils [48] on the thermocouple electromotive force have been made. A more detailed error discussion of temperature measurement is presented in Section 4.1. Some of the samples were homogenised at a higher temperature (*T*_1_ in Table 3) prior to the final annealing step. After homogenisation, these samples were rapidly cooled to the final annealing temperature (*T*_2_ in Table 3) within 5–10 s. After the annealing was finished, all samples were quenched to RT, by switching off the power supply. The cooling rate was sufficiently high to quench the high-pressure state of the sample, e.g., in 14/8 cooling the sample from 1000 °C to 100 °C is accomplished within 10 s and RT is reached after less than 4 min. After the samples reached RT, decompression was initiated. The samples were recovered by breaking the octahedral pressure medium in a vice and dissolving the CsCl capsule in water.

### 2.4. Sample Analysis

The chemical composition of the starting powders was analysed by means of carrier-gas hot-extraction employing a Bruker G8 GALILEO analyser. Three individual measurements were conducted in order to determine average N and O contents as well as their uncertainties. The phase composition of the starting powders was validated by means of XRD (see below).

For microscopic analysis, samples recovered from high-pressure heat treatments were ground and polished along the longitudinal axis of the sample cylinder such that microstructure inhomogeneity due to axial temperature gradients would be discernible. In the final preparation step, the samples were vibration polished for 5 h employing a SiO_2_ polishing agent of 0.02 µm grade. Samples were cold embedded into epoxy resin to avoid potential tempering of the quenched phases.

Microstructure analysis was conducted employing a JSM7800F scanning electron microscope, which was equipped with a field-emission gun. The samples were analysed by means of back scattered electron (BSE) imaging and electron backscatter diffraction (EBSD) during which the microscope was operated at 20 kV. All EBSD datasets were evaluated within the OIM Analysis™ 8 software.

A Bruker D8 ADVANCE diffractometer in Bragg–Brentano para-focusing geometry was used for X-ray diffraction (XRD) analysis. The diffractometer was equipped with a quartz crystal Johannsson incident beam monochromator enabling the use of CoK_α1_ (*λ* = 1.78897 Å) and a Lynxeye™ Si strip position-sensitive detector. Diffraction data were collected in the angular range of 30–140°. The acquired diffraction patterns were processed by the Topas 5 software mostly in terms of Rietveld refinements. Only for samples 2-1000 and 4-1000, which showed strong texture, were lattice parameters determined by Pawley fits. In the refinements Thompson-Cox-Hastings pseudo-Voigt profile functions were employed to describe the Peak shapes. The background was described by a Chebyshev polynomial. To treat systematic errors resulting from sample mounting, a specimen displacement correction was included in the refinements. Lattice parameters reported here are round to the closest multiple of 0.0001 Å, due to the low standard deviations calculated during the refinements (typically below 5 × 10^−5^ Å).

## 3. Results

### 3.1. Analysis of DFT Calculations

**Figure 3:**
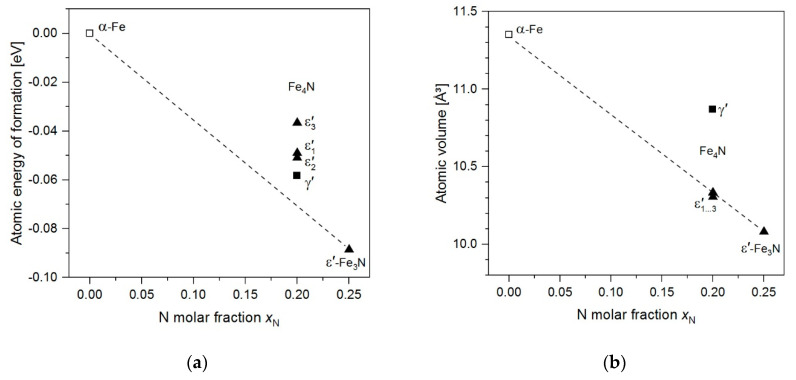
(**a**) Atomic energy of formation from the elements and (**b**) atomic volume (both given per Fe + N atoms) resulting from the DFT calculation for the different Fe-N model structures. The distance of the different data values pertaining to the different Fe_4_N model structures from the lines connecting the values for α-Fe and ε′-Fe_3_N depict the respective (**a**) energy and (**b**) volume of formation values.

The results of the DFT calculations of the model structures are given in Table 2 and energies of formation and atomic volumes are shown in Figure 3. CIF files containing details of the crystal structures of the ε′-Fe_4_N model structures are provided as Supplementary Materials. Note that the structure data were processed using the FINDSYM software [49], whereby standard settings of the space groups were employed, which in the case of ε_2_′-Fe_4_N deviate from the setting chosen in Table 2 to emphasise the relation between the different superstructure unit cells.

Figure 3a illustrates that the energies of formation of all Fe_4_N model structures are located above the line connecting the energies of formation of α-Fe and ε′-Fe_3_N, indicating that Fe_4_N has a positive energy of formation with respect to α + ε′-Fe_3_N, as the distance of energy value of the respective Fe_4_N structure corresponds to corresponding energy of reaction (per atom). These values are included in Table 2, whereby the experimentally known γ′-Fe_4_N structure is that of lowest energy. The atomic volumes shown in Figure 3b show that all the ε′-Fe_4_N model structures have a significantly lower volume than γ′-Fe_4_N, and fall on the line connecting α-Fe and ε′-Fe_3_N.

### 3.2. Analysis of Starting Materials

Carrier gas hot extraction yielded a chemical composition of 5.97(10) wt% N and 0.48(1) wt% O (balance Fe), and thus 20.0 at% N for the pure γ′ powder. Note that the oxygen content includes contributions from adsorbates on the powder particle surfaces. XRD analysis indicated that no oxide phase is present in any of the two initial powders. XRD analysis confirmed that the powder is indeed single-phase γ′ nitride with a lattice parameter of 3.7987 Å. This value corresponds to a N content of 20.0 at% according to Somers et al. [50], which agrees well with chemical analysis.

Carrier gas hot extraction analysis of the α + γ′ powder mixture resulted in 4.30(8) wt% N and 0.41(3) wt% O. This corresponds to an N molar fraction of 15.1 at%. XRD analysis revealed that the powder mixture is composed of 29.5 wt% (26.2 at%) α-Fe with *a* = 2.8669 Å, and 70.5 wt% (73.8 at%) γ′-Fe_4_N with *a* = 3.7989 Å. The average N content calculated from the results of XRD analysis is 14.8 at%, which again is in good agreement with chemical analysis.

### 3.3. Analysis of Phase Constitution and Microstructures of Quenched Samples

Table 4 summarises the primary results from XRD analysis. Fitted XRD patterns and raw data of all samples are provided in the Supplementary Materials. Most of the samples previously heat-treated at high pressure consist of two phases. If the high-pressure high-temperature equilibrium has been successfully retained, such two-phase states correspond to a favourable outcome for phase equilibrium studies in a binary system. Only a few samples are composed of more than two phases. For samples 5-300 and 5-350, this could be ascribed to the low temperatures and insufficient time for equilibration. On the other hand, there are the multi-phase samples 4-450 and 4.5-400 that were heat-treated at *T* ≥ 400 °C, i.e., at temperatures which in other experiments proved to be sufficiently high for equilibration within 4 h. Reasons for the occurrence of three- and four-phase assemblages are discussed more closely in view of the derived phase diagrams in Section 3.4 and *P*–*T* projection in Section 4.1.

**Figure 4:**
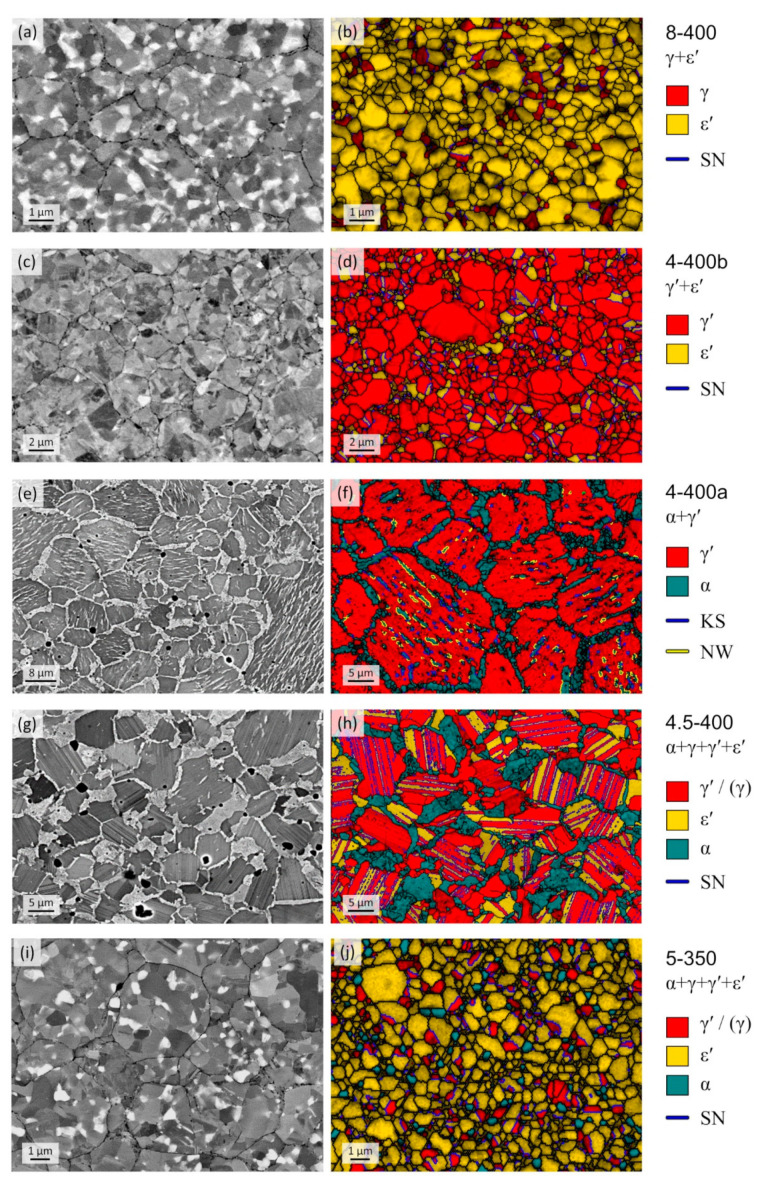
BSE micrographs and EBSD phase maps of samples heat-treated at different pressures and temperatures indicated by the sample notation on the right, e.g., 8-400 meaning 8 GPa and 400 °C. The phases present in the samples as determined by XRD are given below the sample designation. The coloured legend refers to the EBSD phase map. Grain boundaries exhibiting special orientation relationships (Shoji–Nishiyama (SN), Kurdjumov–Sachs (KS) and Nishiyama–Wassermann (NW)) are coloured as indicated to the right of the respective figure.

Figure 4 shows BSE micrographs alongside EBSD phase maps of polished cross sections of selected samples, each representing a unique microstructure recovered after quenching/decompressing from high-pressure/high-temperature to atmospheric conditions. The micrographs are considered to be representative of the entire respective sample as no major spatial variations of the microstructures were observed across a given sample’s cross section. With the exception of sample 4.5-400, shown in Figure 4g,h, which exhibits a large number of planar interfaces, none of the samples showed microstructure features that would be indicative of diffusionless phase transformations, which might have occurred during quenching or decompression. Such phase transitions generally involve mechanisms, which result in microstructures with high defect density and occasionally very small grain sizes of the product phase. In XRD patterns, this typically manifests in strongly broadened (due to involved defects) or even split XRD reflections (if symmetry is reduced) of the product phase. A prominent example is the martensitic transformation fcc γ-Fe(N) → bct α′-Fe(N), which was not observed in any of the samples. In fact, the N content determined for the γ-Fe(N), which formed in some of the samples (see Table 5), is larger than 8.2 at%, and is thus high enough to facilitate metastable retention of the γ-Fe(N) at RT and atmospheric pressure [51]. It can generally be concluded that Fe–N phases, in the composition region Fe–Fe_3_N having a N content of 8–9 at%, can be quenched/decompressed from high-pressure/high-temperature conditions.

The microstructures of samples 8-400 and 4-400b shown in Figure 4a–d have in common that they were generated from pure γ′ powder. The phases in these microstructures are composed of phases having fcc and hcp Fe sublattices, i.e., γ + ε′ and γ′ + ε′, respectively. It should be noted that the fcc-based γ and γ′ phases cannot be distinguished by means of EBSD analysis, as the superstructure bands of the ordered γ′ phase were indiscernible, and the employed indexing software did not account for Kikuchi bandwidths. However, the two phases be easily distinguished by means of XRD analysis: In the XRD pattern of sample 4-400b, superstructure reflections of the γ′ phase are clearly visible (see Supplementary Materials) and, in addition, the fundamental reflections of the γ′ phase are shifted to significantly lower diffraction angles due to its larger lattice parameter as compared to γ. In some of the samples, e.g., sample 2-500, containing the γ′ phase, superstructure reflections are not visible in the XRD patterns. However, the markedly large lattice parameter and narrow homogeneity range still suffice for unambiguous identification of the γ′ phase.

In both microstructures shown in Figure 4a–d, an orientation relationship ${\left\{111\right\}}_{\gamma /\gamma^{\prime }}∥{\left\{0001\right\}}_{\varepsilon^{\prime }}$, ${\langle 1\bar{1}0\rangle }_{\gamma /\gamma^{\prime }}∥{\langle 10\bar{1}0\rangle }_{\varepsilon^{\prime }}$ is frequently observed. The same observation was made in our previous study [32] and other works [52,53] that investigated iron carbonitride materials. This orientation relationship is equivalent to the so-called Shoji–Nishiyama (SN) [54] orientation relationship ${\left\{111\right\}}_{\mathrm{fcc}}∥{\left\{0001\right\}}_{\mathrm{hcp}}$, ${\langle 1\bar{1}0\rangle }_{\mathrm{fcc}}∥{\langle 11\bar{2}0\rangle }_{\mathrm{hcp}}$, if a hcp unit cell is adopted for the Fe atoms in the ε′ nitride. Aside from this, the microstructures are characterised by small, globular grains.

It should be noted that the formation of γ′ + ε′ phase equilibria from the γ′ initial powder, as observed for sample 4-400b, was unexpected at first. However, the formation of a γ′ + ε′ equilibria from single-phase γ′ samples is quite reasonable: Vacancy formation on the otherwise perfectly ordered N sublattice of γ′ increases configurational entropy and reduces molar volume, and it is thus likely that the γ′ phase adopts an equilibrium N content ≤20 at% at both elevated temperature and pressure. This is supported by the determined lattice parameters (as low as 3.7912 Å, see Table 4), which are smaller for γ′ in equilibrium with ε′ than for the γ′ in the initial powder. Further, it is evident that the lattice parameter of the γ′ phase in equilibrium with ε′ decreases with increasing pressure at constant temperature. Oxide formation, which also could cause local enrichment in N and thus ε′ nitride formation in the vicinity of oxide particles, is excluded here as no indication of iron oxide could be seen in the diffraction patterns of any of the γ′ samples.

The samples 4-400 and 4.5-400 shown in Figure 4e–h have both been subjected to a two-step heat treatment. Starting from the initial α + γ′ mixture, the samples were heated up to 1000 °C for 0.5–1 h before they were rapidly cooled to 400 °C and annealed for a further 4 h. It has been shown for samples 2-1000 and 4-1000 (see Table 4) that the heat treatment for 0.25 and 1 h at 1000 °C results in the formation of single-phase ε′ nitride and thus facilitates homogenisation. As a result of the initial homogenisation step, the microstructures of the respective samples are more coarse-grained than of samples heat-treated at 400 °C only, and need to be interpreted to be the result of a phase transformation starting from the ε′ phase.

The microstructure of sample 4-400a shown in Figure 4e,f is composed of α + γ′ as also implied by XRD analysis. The microstructure clearly shows two different morphologies of the α phase which are reminiscent of morphologies of ferrite (α solid solution) in hypo-eutectoid Fe–C alloys [55]. The first corresponds to allotriomorphic ferrite, i.e., α grains which form seams around former ε′ grains (cf. γ-Fe(C) in Fe–C) and the second to intragranular ferrite in the interior of former ε′ grains. A striking feature of the microstructure in Figure 4e,f is the parallel arrangement of the intragranular ferrite plates. The γ′ grains are frequently intersected by Σ3 twin boundaries. Most of the intragranular ferrite plates exhibit {110} planes that are parallel to the {111} Σ3 twin plane of the γ′ phase. In particular, orientation relationships that are near Kurdjumov–Sachs (KS) or near Nishiyama–Wassermann (NW) and intermediate were observed (more detailed investigation was beyond the scope of the present work). Three to six orientation variants of the α phase were observed per γ′ grain. The apparent dominance of α plates with ${\left\{110\right\}}_{\alpha }$ parallel with *one* particular ${\left\{111\right\}}_{\gamma^{\prime }}$ is likely the result of bcc nucleation from the ε′ nitride, which formed during the initial 1000 °C heat treatment. Due to the hcp-like arrangement of Fe atoms, grains of ε′ exhibit only one set of close-packed planes that can be involved in the displacive hcp → bcc transformation of the Fe sublattice, which is in accordance with the observed orientation relationship. The ε′, having an average N content of 15 at% after the homogenisation treatment, most likely transformed into γ′ upon N partitioning between α and surrounding ε′ again involving a diffusional-displacive transformation. A high number of Σ3 twin boundaries remain as a trace of the hcp → fcc transformation. Another explanation for the low number of bcc variants could be a variant selection due to the presence of non-hydrostatic stress. Similar microstructures have been observed for Widmanstätten ferrite in hypo-eutectoid steels which were heat-treated under uniaxial compression [56]. Non-hydrostatic stresses are likely to occur in high-pressure experiments with solid pressure media and represent a potential cause of a varying number of variants per γ′ grain.

The micrographs of sample 4.5-400 shown in Figure 4g,h are most indicative of the displacive nature of the phase transformations in the Fe–N system. This sample exhibits a microstructure with straight-line phase boundaries between the fcc-like γ′ and hcp-like ε′, a feature which was only observed in samples that have been homogenised in the ε′ phase region in the first annealing step. Again, an SN orientation relationship is commonly observed at these phase boundaries, and the fcc grains are intersected by Σ3 twin boundaries. According to XRD, sample 4.5-400 is mainly composed of the three phases α, γ′, and ε′, as well as minor amounts of γ solid solution (see Table 4). In the EBSD analysis α, ε′, and fcc-type phases γ/γ′ could be identified. The α phase grains predominantly exhibit a morphology that is comparable with allotriomorphic ferrite in hypo-eutectoid steels. In contrast to sample 4-400, only few acicular shaped α grains can be seen in the ex-ε′ grain interiors (see Figure 4g). In view of the high phase fraction of γ′ in this sample determined in XRD analysis (see Table 4), it is concluded that most of the grains sharing planar interfaces with the ε′ phase correspond to the γ′ phase.

Figure 4i,j shows the microstructure of sample 5-350. As shown by XRD, the sample is composed of four phases (see Table 4), which have formed from single-phase γ′ during a 4 h heat treatment at 5 GPa and 350 °C. In contrast to sample 4.5-400, sample 5-350 has not been homogenised at 1000 °C, as the initial powder was already composed of homogeneous γ′ nitride. The microstructure of sample 5-350 is characterised by globular grains and only a small number of planar phase boundaries between ε′ and γ-type phases. Again, ε′ + γ/γ′ phase boundaries exhibit the typical SN orientation relationship. It is further important to note that this is the sample of the lowest temperature in which the γ terminal solid solution phase has formed. Due to the single-step heat treatment, the γ phase present in the sample cannot be a remainder of a high-temperature annealing step in which, due to the presence of a γ + ε′ two-phase region, minor amounts of γ could potentially have formed. Instead, the presence of small amounts of γ solid solution points to its stabilisation from 592 °C at atmospheric pressure [7] to ≥ 350 °C at 5 GPa. A detailed discussion of the reasons for the occurrence of three or four phases is presented in Section 4.1, taking the evolution of phase equilibria with increasing pressure into account.

In order to determine the equilibrium N contents *x*_N_ of the phases, the following relationships of unit cell volume or lattice parameters on N content were employed: For the α terminal solid solution, unit cell volume data of quenched N-supersaturated α′ solid solution (martensite), extrapolated to N-free α-Fe were adopted [57]. For the γ solid solution phase, volume data of quenched N-austenite were used [57]. The lattice parameter data published by Somers et al. [50] were employed for the determination of the N content of γ′ nitride. The resulting N contents are given in Table 5.

**Figure 5:**
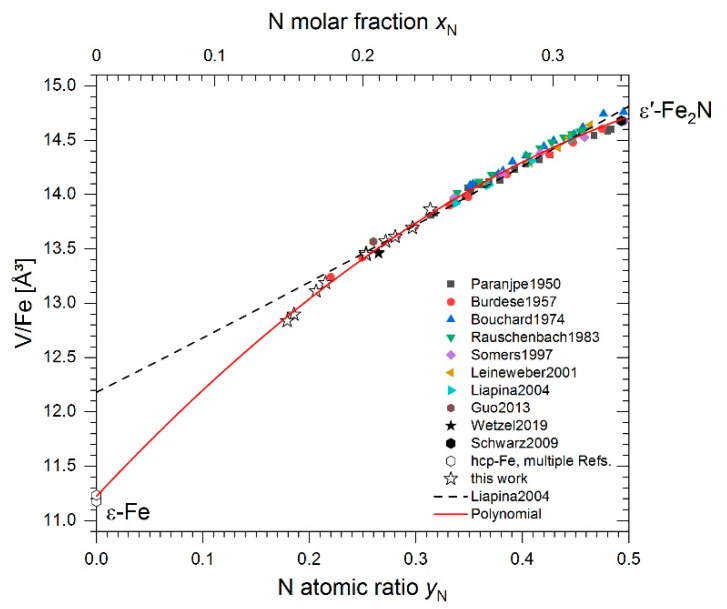
Plot of volumes per Fe atom for ε′ nitrides of various N contents as a function of the ratio of N atoms per Fe atom $y_{N}$. Literature data are collected from References [5,28,32,58,59,60,61,62,63,64]. Data for (N-free) ε-Fe correspond from extrapolation of equations of state published in [65,66,67]. Own data are depicted by star symbols. Note that only data of single-phase ε′ samples and γ + ε′ samples are shown. The red line shows the second-degree polynomial (see Equation (2)) fitted to the data. The black dashed line shows the $y_{N}$ dependence of the volume derived by Liapina et al. [58], which has been used for N content determination in our previous work.

N contents of the ε′ phase were initially calculated from the experimentally determined unit-cell volumes using the reported lattice parameters *a* and *c* of quenched ε′ nitrides [58]. This, however, led to unexpectedly small N contents of 11.5 and 11.4 at% (cf. Figure 5) for samples 2-1000 and 4-1000, respectively. This would imply that these samples have lost significant amounts of N during heat treatment. However, composition analysis of several other samples by means of phase composition and lattice parameters confirmed that, typically, no N loss had occurred during the high-pressure heat treatments. In sample 4-400a, for example, the phases α + γ′ formed in the same proportions as were present in the initial powder. This sample had been homogenised for 1 h at 1000 °C prior to the 4 h 400 °C step, making it comparable to sample 4-1000 which was heat-treated for 1 h at 1000 °C. Calculating the average N content of sample 4-400a from the XRD phase fractions and the N contents of the phases α and γ′ (which can also be approximated to 0 and 20 at%, respectively), a value of 15.2 at% was obtained, which is consistent with the N content of the initial powder mixture. Another example is sample 2-600, where a transformation $\gamma^{\prime }\to \varepsilon^{\prime }$ was realised. The product, supposedly ε′-Fe_4_N, has a unit cell volume of 80.76 Å^3^, which agrees well with literature data for ε′-Fe_4_N typically ranging from 80.53 Å^3^ [59] to 81.40 Å^3^ [60]. In our previous work [32], an almost identical value of 80.80 Å^3^ has been obtained for ε′-Fe_4_N, which had formed during a heat treatment for 1 h at 13 GPa and 880 °C. This shows that samples heat-treated at quite different *P*–*T* conditions yield crystallographic data, which comply with the preservation of the average N content of the samples. Thus, it is concluded that previously reported volume–composition relationships for the ε′ phase typically derived for compositions Fe_3_N_0.75…1.5_ should not be employed to estimate N contents of ε′ nitride with N contents smaller than 20 at% N.

Instead, an alternative volume–composition relationship has been derived, in order to determine a consistent set of N contents for any of the ε′ phases in the entire composition range required. Having ruled out the potential N loss during heat treatments, the XRD data of the dual-phase γ + ε′ and single-phase ε′ samples were used to derive such a relationship. The N content of single-phase ε′ samples was taken as the N content of the initial powders determined from chemical analysis whereas the N content of the ε′ phase of two-phase samples was calculated utilising the lever rule

(1) $$w_{N}^{\varepsilon^{\prime }}=w_{N}^{\gamma }+\left(w_{N}-w_{N}^{\gamma }\right)/w^{\varepsilon^{\prime }},$$

where $w_{N}$ is the total N mass fraction of the initial powders, $w_{N}^{\varepsilon^{\prime }}$ and $w_{N}^{\gamma }$ are the mass fractions of N in the ε′ and γ phases, respectively, and $w^{\varepsilon^{\prime }}$ is the mass fraction of the ε′ phase as determined in XRD analysis. The resulting data are listed in Table 5 and shown in Figure 5 alongside various literature data. It can be seen that the volumes of ε′ nitride with $y_{N}<0.2$ deviate markedly from the relationship derived by Liapina et al. [58] for quenched high-N ε nitrides. Instead, the data seem to be described appropriately by a second-degree polynomial, which extrapolates to the atomic volume of pure ε-Fe

(2) $$V_{\mathrm{Fe}}/{Å}^{3}=11.22\left(2\right)+10.49\left(15\right)\;y_{N}-7.01\left(26\right)\;y_{N}^{2},$$

where $V_{\mathrm{Fe}}$ is the volume per Fe atom and $y_{N}$ is the N atomic fraction, i.e., the number of N atoms per Fe atom.

More detailed considerations on the physical origin of the nonlinear composition dependence of the volumes of ε-Fe(N) and ε′ iron nitrides on N content will be reported in a subsequent paper. Equation (2) has been used to determine the N contents of the ε′ phase which are listed in Table 5, and were used to construct the phase diagrams presented in Section 3.4.

Similar to sample 4-400a discussed earlier, average N contents of any of the other samples have been calculated from the N contents determined from lattice parameters and phase fractions to check the results for consistency. As a result, average N contents of 20.0–20.5 at% were calculated for samples that started from a pure γ′ powder, and values of 14.7–15.7 at% N were obtained for samples starting from a α + γ′ powder mixture. These values agree well with the average N contents of the initial powders, indicating the consistency of XRD phase fractions and N contents of the phases, and proving the validity of the approach.

### 3.4. Phase Equilibria in the Fe-Rich Part of Fe–N System at High Pressure

**Figure 6:**
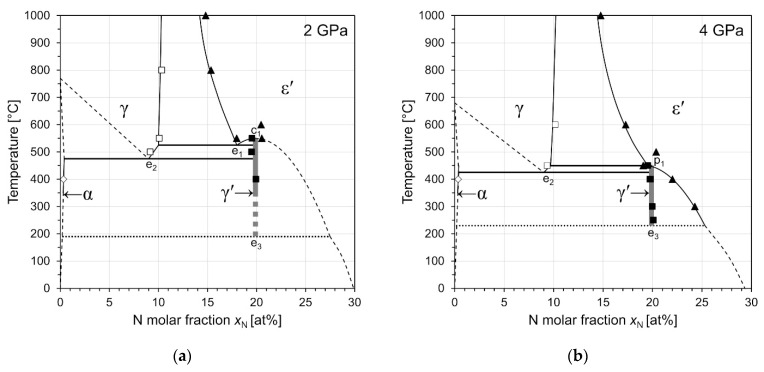
Partial temperature–composition phase diagrams of the Fe-rich part of the system Fe–N at (**a**) 2 GPa and (**b**) 4 GPa. Due to its narrow homogeneity range, the phase region of the γ′ phase has been displayed as thick solid line.

**Figure 7:**
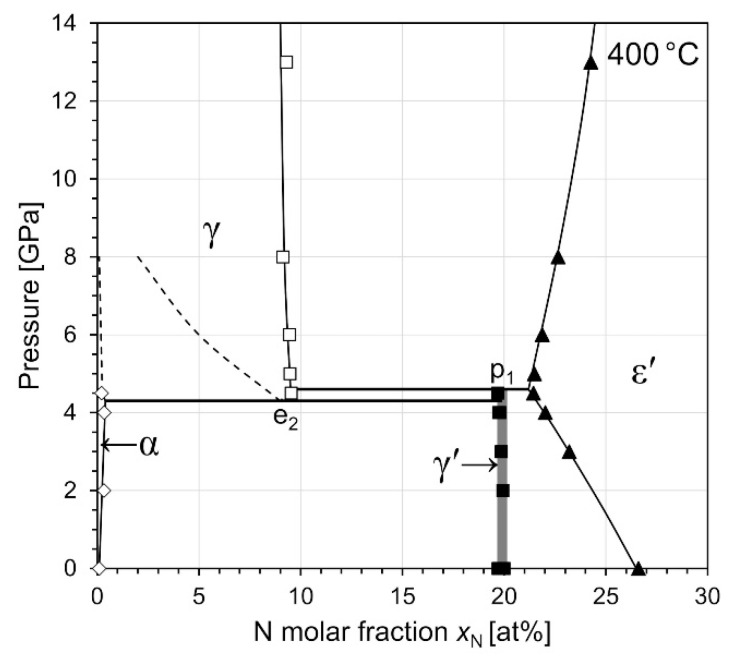
Pressure–composition diagram of the phase equilibria at 400 °C. Data at 1.01 × 10^−5^ GPa are calculated from a thermodynamic database [7] setting the N_2_ gas phase dormant. The two data points at 13 GPa are taken from our previous work [32].

According to the results presented so far, interpretation of the experimental phase composition data in terms of phase equilibria attained at the high-pressure heat treatment conditions is considered appropriate: All samples were rapidly quenched to RT, and successively decompressed, which ensured that both phase composition and N content of the phases were retained. Evidently, phase formation and N diffusion is typically sufficiently fast at *T* ≥ 400 °C to allow attaining an equilibrium state within 4 h of heat treatment. In addition, microstructure analysis showed no indications of martensitic transformations and thus confirms that the studied phases are quenchable. Hence, the constitution data given in Table 5 were used to construct partial isobaric temperature–composition (*T*–*x*) diagrams for pressures 2 and 4 GPa (see Figure 6) as well as an isothermal pressure–composition diagram at 400 °C (see Figure 7).

#### 3.4.1. Isobaric Sections at 2 GPa and 4 GPa

Figure 6a shows a partial temperature–composition diagram of the Fe–N system at 2 GPa. The data suggest that the congruent reaction $\varepsilon^{\prime }⇌\gamma^{\prime }$ (c_1_) is still persistent at this pressure. This detail is in contrast to the estimated *P*–*T* projection of our earlier work [32]. Two equilibria γ + ε′ and γ′ + ε′ formed in samples 2-550a and 2-550b from starting powders containing 15 and 20 at% N, respectively. The ε′ phase in these two samples exhibits clear differences in the lattice parameters (see Table 4), such that the γ′ + ε′ equilibrium is constituted by ε′ with larger lattice parameters, and thus higher N content. The fact that these two distinct equilibria were formed at 550 °C, and that single-phase ε′ was obtained at 600 °C for sample 2-600, indicates that reaction c_1_ is shifted from 691 °C at atmospheric pressure [7] to a temperature between 550 and 600 °C at 2 GPa. Further, it can be concluded that the eutectoid reaction $\varepsilon^{\prime }⇌\gamma +\gamma^{\prime }$ (e_1_) is located between 550 and 500 °C, as in sample 2-500 a dual-phase equilibrium of γ + γ′ was formed. Sample 2-400 is composed of α + γ′, which indicates that the eutectoid reaction $\gamma ⇌\alpha +\gamma^{\prime }$ (e_2_) must be located between 400 and 500 °C at 2 GPa, i.e., much lower than anticipated in our previous work [32].

The phase equilibria assessed for 4 GPa are depicted in Figure 6b. In contrast to the situation at 2 GPa, the ε′ phase boundary of the γ + ε′ region at high temperature is shifted towards higher N contents. As a result, the N contents of the γ′ phase and the ε′ phase are virtually identical at 4 GPa and 450 °C, and sample 4-450, apart from the γ phase, contains both γ′ and ε′ nitride phases in substantial quantities. It is assumed that the conditions of the high-pressure heat treatment were very close to the coincidence of reactions c_1_ and e_2_. That coincidence would result in the emergence of a new reaction, $\gamma +\varepsilon^{\prime }⇌\gamma^{\prime }$ (p_1_), as already predicted in our previous work [32]. Equal N contents of the ε′ and γ′ phases, however, render reaction p_1_ compositionally degenerate, i.e., it cannot be distinguished from c_1_ or e_1_. An alternative explanation could be that the time of the heat treatment did not suffice for full equilibration in this sample. This option is excluded as, for other samples heat-treated at 400 °C, it could be shown without any doubt that a two-phase assemblage can be formed within 4 h of heat treatment. The potential occurrence of a diffusionless transformation $\varepsilon^{\prime }\to \gamma^{\prime }$ during quenching/decompression is also considered unlikely as the ε′ phase has proven to be quenchable for various compositions in many other samples (e.g., 4-500 and 4-1000).

In contrast to sample 4-450, the interpretation of the other equilibria formed at 4 GPa is more straightforward: In sample 4-400a, i.e., at 4 GPa and 400°C, an α + γ′ equilibrium has formed. This shows that reaction e_2_ is located between 400 and 450 °C, and that p_1_ and e_2_ are very close to one another. A single-phase γ′ sample was heat-treated at similar conditions in experiment 4-400b, which resulted in the formation of a γ′ + ε′ equilibrium. The lattice parameter of the γ′ phase (*a* = 3.7957 Å) indicates that it attained a slightly lower equilibrium N molar fraction as compared to the γ′ initial powder (*a* = 3.7987 Å), which explains the formation of a small amount of additional ε′ phase in this sample. Similar results are obtained for sample 4-300 and in sample 4-250, where only very faint reflections of an additional phase, most likely ε′ are visible in the XRD pattern (see Supplementary Materials).

#### 3.4.2. Isothermal Section at 400 °C

Figure 7 shows an isothermal *P*–*x* diagram constructed from all phase constitution data determined from samples heat-treated at 400 °C. In our previous study [32] the decomposition of γ′ into γ + ε′ at 13 GPa and 400 °C has been observed. The N contents of γ and ε′ phase at 13 GPa (also included in Figure 7) are in good agreement with the trend of the present data. It can be seen that the γ + ε′ equilibrium, which results from the retraction of the γ′ phase region below the 400 °C plane, occurs already at a pressure as low as 5 GPa. The γ′ + ε′ equilibria observed at 3 and 4 GPa were formed from single phase γ′ samples. The highest pressure at which the γ′ phase was still observed at 400 °C is 4.5 GPa. At these conditions all four phases α, γ, γ′, and ε′ have formed. Due to the presence of four phases in sample 4.5-400, with only minor amounts of the γ solid solution, the transition pressure of the reaction $\gamma ⇌\alpha +\gamma^{\prime }$ (e_2_) reaction cannot be determined unambiguously. However, it can be concluded that the intersection of reaction e_2_ with the 400°C plane occurs between 4 and 5 GPa. The formation of γ′ phase at 4.5 GPa and its decomposition at 5 GPa into γ + ε′ indicates that the reaction $\gamma +\varepsilon^{\prime }⇌\gamma^{\prime }$ (p_1_) intersects with the 400 °C plane between 4.5 and 5 GPa. This, in turn, implies that the γ′ phase could still be stable at *T* < 400 °C at 5 GPa, as will be discussed more closely in Section 4.1. Note that in contrast to sample 4-450, the ε′ phase now has an N content which is clearly different from that of the γ′ phase, demonstrating the peritectoid character of the reaction.

It is important to note that the α + γ′ equilibria at 2 and 4 GPa formed from α + γ′ powder mixtures *after* having been homogenised for 1 h in the ε′ phase region at high temperature. This means that those samples were subject to a series of phase transitions $\alpha +\gamma^{\prime }\to \varepsilon^{\prime }\to \alpha +\gamma^{\prime }$, which proves that γ′-Fe_4_N can be *reversibly* formed at high-pressure/high-temperature conditions from a phase (ε′) which has a lower molar volume. This indicates that γ′ is very likely thermodynamically stable at 4 GPa and 400 °C, and that the actual phase equilibria are not obscured by metastable retention of low-volume phases.

#### 3.4.3. Low-Temperature Eutectoid Decomposition of γ′-Fe_4_N

In our previous work [32] it has been calculated that the equilibrium temperature *T*_0_ of the eutectoid decomposition reaction $\gamma^{\prime }⇌\alpha +\varepsilon^{\prime }$ (e_3_) will rise with increasing pressure, irrespective of its actual value of *T*_0_ at atmospheric pressure. Due to these expectations, a series of low-temperature heat treatments were conducted in order to find direct experimental evidence for the eutectoid decomposition reaction e_3_.

In samples 4-250 and 4-300, the γ′ phase is still retained, and only minor amounts of additional ε′ phase have formed. This is in accordance with the γ′ + ε′ equilibrium, which has been observed for other conditions, too (e.g., samples 3-400 and 4-400). In contrast to sample 4-300, sample 5-300 contains both the α and the ε′ phase alongside the γ′ nitride. This indicates that a partial transformation $\gamma^{\prime }\to \alpha +\varepsilon^{\prime }$ has been realised. It is thus evident that reaction e_3_ is located above 300 °C at 5 GPa and below 300°C at 4 GPa. Due to absence of the α phase in sample 4-250, it is supposed that reaction e_3_ is located below 250 °C at 4 GPa, although it remains arguable whether the transformation is just kinetically impeded, resulting in the metastable retention of the γ′ phase.

The fact that sample 5-300 still contains γ′ phase alongside α + ε′ is considered to be a result of incomplete transformation due to the low transformation rate at that temperature. For sample 5-350, this explanation is not satisfactory as *four* phases have formed at an even higher temperature. On contrary, at 5 GPa and 400 °C only (sample 5-400) only the two-phase equilibrium γ + ε′ is observed. It is thus supposed that the heat-treatment conditions of sample 5-350 might be close to a four-phase equilibrium, as will be discussed more closely in the following.

## 4. Discussion

### 4.1. Evolution of the Phase Equilibria in the Fe–N System Subjected to High Pressure

In this section, the phase equilibrium data presented above will be used to derive a reaction path for the pressure induced disappearance of the γ′ phase, which will represent the experimental basis for the discussion of the pressure dependent phase stability in the Fe-rich part of the Fe–N system up to a pressure of 8 GPa.

**Figure 8:**
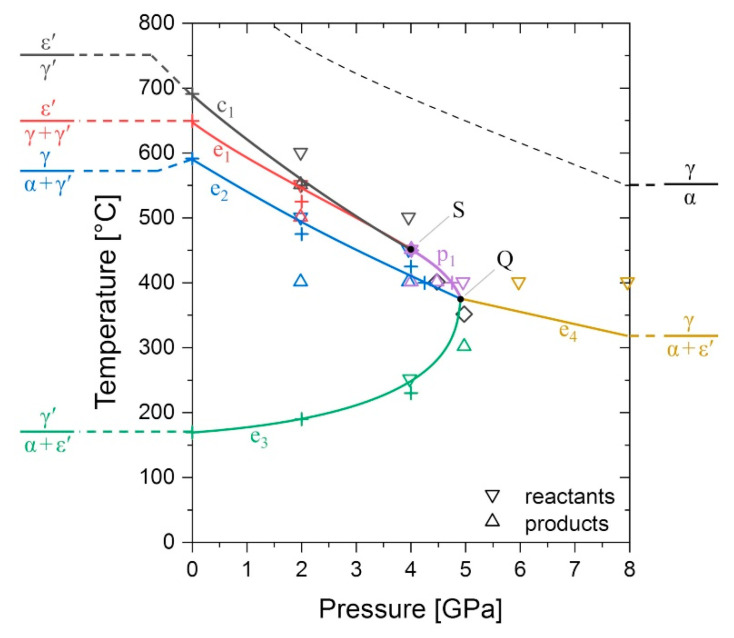
*P*–*T* projection of the univariant equilibria in the Fe-rich part of the Fe–N system, derived from experimental equilibrium data. Triangles facing up or down show experimental data of products and reactants, respectively, diamonds indicate conditions in which four phases were present, and plus signs indicate the conditions of three-phase equilibria presented in Figure 6 and Figure 7. Data at 1 × 10^−4^ GPa were calculated from the thermodynamic database of Göhring et al. [7]. Lines show the pressure-dependent evolution of the univariant equilibria in the system. The dashed line shows the equilibrium conditions for allotropic transition α⇌γ calculated with the database of Lu et al. [68]. The labels indicate the phases participating in the respective univariant equilibria. Phase labels written above and below the line represent phases stable at temperatures higher and lower than indicated by the univariant line.

Figure 8 shows a projection of the univariant equilibria in the system Fe–Fe_3_N into the *P*–*T* plane. Solid lines show the evolution of equilibria corresponding to the congruent reaction $\varepsilon^{\prime }⇌\gamma^{\prime }$ (c_1_), the eutectoid reactions $\varepsilon^{\prime }⇌\gamma +\gamma^{\prime }$ (e_1_), $\gamma ⇌\alpha +\gamma^{\prime }$ (e_2_), $\gamma^{\prime }⇌\alpha +\varepsilon^{\prime }$ (e_3_), $\gamma ⇌\alpha +\varepsilon^{\prime }$ (e_4_), and a peritectoid reaction $\gamma +\varepsilon^{\prime }⇌\gamma^{\prime }$ (p_1_). The *P*–*T* projection additionally features a singular point S and a quadruple point Q, which will be discussed in more detail in the following.

The data obtained from heat treatments at 2 GPa clearly show that both reactions c_1_ and e_1_ persist at this pressure. Based on sample 4-450, in which γ′ and ε′ phases of nearly identical N content coexist with the γ solid solution, it is concluded that the reactions c_1_ and e_1_ coincide at approximately 4 GPa and 450 °C. Due to the presence of only two phases with equal composition (instead of three phases), the ε′ + γ′ equilibrium of c_1_ is singular. The univariant line of this singular equilibrium ends asymptotically in the point S (close to 4 GPa and 450 °C) and the three-phase line of γ + ε′ + γ′ continues to higher pressures [69]. At the point S there is a transition of the γ + ε′ + γ′ equilibrium from a eutectoid $\varepsilon^{\prime }⇌\gamma +\gamma^{\prime }$ (e_1_) at *P* < 4 GPa to a peritectoid type $\varepsilon^{\prime }+\gamma ⇌\gamma^{\prime }$ (p_1_) at *P* > 4 GPa. Further, the γ + γ′ + ε′ equilibrium is compositionally degenerate at that point. In view of Figure 1, it is obvious that coincidence of reactions c_1_ and e_1_ must lead to compositional degeneracy for e_1_/p_1_, as the formerly compositionally distinct state points of the ε′ phases in c_1_ and e_1_ will then be identical and coincide with that of the γ′ phase. This, in turn, imposes the constraint of identical Clapeyron slopes d*T*/d*P* of c_1_/e_1_/p_1_ in the *P*–*T* projection at the point of intersection.

As has been stated earlier, samples 4.5-400 and 5-350 are composed of four phases. A possible explanation for the coexistence of four phases could be the existence of temperature and pressure gradients in the high-pressure cell. However, microscopic analysis of multiple different locations in these samples did not reveal any marked variation in the microstructure, implying that large temperature or pressure gradients in the sample are not the leading cause of the presence of four phases. At this point, it should be mentioned that for sample 4.5-400 phase composition did vary slightly in XRD measurements between top, bottom, and the polishing plane, but reflections due to any of the four phases were visible in all the measurements. Another explanation for the occurrence of four phases could be the presence of local equilibria, resulting from the transformation of two distinct phases in a formerly inhomogeneous sample. This interpretation is also excluded as the α phase has common interfaces with both γ′ and ε′ in the two samples.

According to Gibbs’ phase rule, four phases can coexist in equilibrium in a binary system at a quadruple point at a specific pressure and temperature, but it is rather unlikely that the exact conditions for coexistence are met during an experiment. Instead, it is supposed that the *P*–*T* conditions chosen for the treatments of the two samples, are close to a quadruple point in the Fe–N system (see Figure 8) leading to a situation where all four phases (especially γ′ and ε′) have a low difference in Gibbs energy, and thus coexist metastably. In other words, any of the univariant equilibria p_1_, e_2_, and e_3_ are very close to one another in the pressure range 4.5–5 GPa and temperature range 400–350 °C. It is expected that a quadruple point Q exists within the given *P*–*T* range. In the simplest case shown in Figure 8, reactions p_1_, e_2_, and e_3_ would coincide in the quadruple point leaving a new eutectoid reaction $\gamma ⇌\alpha +\varepsilon^{\prime }$ (e_4_) on the high-pressure side of Q. Reaction e_4_ was already predicted in our previous work [32] and is considered the only reasonable univariant reaction involving α, γ, and ε′, as γ-Fe is the high temperature allotrope of Fe. No further attempt to verify the existence of reaction e_4_ has been made in the current study.

The coincidence of reactions p_1_, e_2_, and e_3_ in an invariant point results in the disappearance of the γ′ phase from the system at a pressure above that point. Alternatively, there are two other conceivable scenarios for the disappearance of the γ′ phase at high pressure: (i) constriction of the γ′ phase region in the γ + ε′ region (at temperature above point Q) or (ii) constriction of the γ′ region in the α + ε′ region (at temperatures below Q). Scenario (i) has already been discussed in our previous paper [32] but is now, given the experimental data presented here, considered unlikely. Scenario (ii) requires another peritectoid reaction $\alpha +\varepsilon^{\prime }⇌\gamma^{\prime }$ (p_2_) originating from the quadruple point with p_1_, e_2_, and e_4_. With increasing pressure reaction, p_2_ is shifted towards lower temperatures and, together with e_3_, results in the constriction of the γ′ phase region. The constriction of the γ′ region by two α + γ′ + ε′ equilibria with opposite signs of the Clapeyron slopes requires the existence of a second singular point with infinite slope d*T*/d*P*. In view of the steep slope of reaction e_3_ close to point Q in Figure 8 scenario (ii) appears more reasonable. However, a slope d*T*/d*P* = Δ*V*/Δ*S* → ∞ implies Δ*S* → 0, a thermodynamic coincidence that cannot be excluded but is considered rather unlikely.

The different scenarios for the disappearance of the γ′ phase are expected to occur in such a small *P*–*T* window, which, within the limits of experimental error, renders their distinction difficult. The data at 2 GPa indicate that reactions c_1_, e_1_, and e_2_ are shifted towards low temperatures to an unexpected extent. In our previous work a Clapeyron slope of −25 K/GPa has been estimated for the univariant line of e_2_. In contrast, a value of −45 K/GPa is obtained using the experimental data of the present study. It should be mentioned that the Clapeyron slope calculations are quite sensitive to the somewhat ambiguous thermal expansion behaviour of the ferromagnetic nitride phases. The γ′ nitride, for example, is expected to show an Invar-like thermal expansion behaviour [70], typically resulting in a higher thermal expansion coefficient at temperatures above the Curie temperature. However, there exists no data of the thermal expansion coefficient above its Curie point. Hence, the volume of that phase used in our previous calculations [32] could be underestimated by extrapolation of the thermal expansion to high temperatures.

The main uncertainty, however, should be attributed to temperature measurement errors which arise due to thermal gradients in the pressure cells. The CsCl, which has been used as a capsule material in this study, has a low thermal conductivity compared to the other parts in the pressure cell. The resulting thermal gradient along the capsule wall can lead to an offset such that the temperatures read from the thermocouple are generally lower than the actual temperatures in the samples. Using the software provided by Hernlund et al. [71] we estimate that the temperature data reported here can indeed be in the order of 10–15% lower than the actual sample temperatures for the two geometries applied. However, it should be noted that this offset is systematic and, therefore, should not affect the general trend of the data, if care is taken to keep experimental conditions comparable. This is supported by the smooth trends in the phase boundary data shown in Figure 7.

In the current study, pressure estimation has been limited to the determination of external pressure calibration curves at RT, which represents another source of error. During heating, the pressure in the sample can increase due to suppression of its thermal expansion and, at the same time, pressure can be reduced by the decrease in flow strength of the pressure medium and gaskets. If the second contribution is neglected and heating is assumed to be fully isochoric, the thermal pressure can be estimated according to Δ*P*_th_ = *αK*Δ*T*, where the volume thermal expansion coefficient *α* and bulk modulus *K* are assumed to be constant in pressure and temperature. Using *α* = 143 × 10^−6^ K^−1^ [72] and *K* = 16.7 GPa [73] for CsCl, and *α* = 22.9 × 10^−6^ K^−1^ [50] and *K* = 156 GPa [60] for γ′-Fe_4_N, thermal pressure coefficients of 2.39 × 10^−3^ and 3.57 × 10^−3^ GPa/K are obtained assuming that the sample is composed entirely of CsCl or Fe_4_N, respectively. This results in an average thermal pressure Δ*P*_th_ ≈ 1.1 GPa at 375 °C, which is considered to be an upper estimate of the pressure error.

Taking into account the present experimental data and the errors discussed above, it is concluded that the γ′-Fe_4_N phase vanishes from the Fe–N system at a pressure of $5.0_{-0.5}^{+1.1}\;\mathrm{GPa}$ and a temperature of $375_{-25}^{+56}$ °C.

### 4.2. Discussion of the P–T-Dependent Phase Stability

In this section, the phase stability of phases relevant for the Fe-rich portion of the Fe–N system will be discussed, taking into account literature data and results present DFT calculations on the temperature-dependent phase stability in the condensed Fe–N system at atmospheric pressure. The stability trend reflected at atmospheric pressure will then be extended to the high-pressure situation.

Low-temperature stability of Fe–N phases has been covered already much earlier by many other works. A good overview is given by du Marchie van Voorthuysen et al. [10]. In the following, only those works that are considered most relevant, will be summarised. Malinov et al. [9] conducted extensive low-temperature heat treatments of samples of different N content and phase composition. In that work, the γ′ phase has formed at any of the investigated temperatures between 100 and 200 °C. It has been concluded that γ′-Fe_4_N is a stable phase at temperatures as low as 100 °C at atmospheric pressure. To our knowledge, the most recent publication devoted to the eutectoid decomposition reaction $\gamma^{\prime }⇌\alpha +\varepsilon^{\prime }$ (e_3_) is represented by Reference [10]. In that work, a low-temperature extension of the Fe–N phase diagram has been approached by investigation of phase formation during a special low-temperature nitriding process. Thereby, a temperature of 214 °C for the α + γ′ + ε′ equilibrium at atmospheric pressure has been estimated from extrapolations of phase boundary data. However, neither of the two works (and References therein) reported a decomposition of the γ′ phase into α + ε′. Thus, the partial transformation $\gamma^{\prime }\to \alpha +\varepsilon^{\prime }$ observed in the present work (sample 5-300) represents the first direct observation of this reaction.

The instability of γ′-Fe_4_N with respect to α + ε′ at ambient pressure and sufficiently low temperature is well reflected by first-principles calculations taking the ε′-Fe_3_N model structure as representative for the non-stoichiometric ε′ phase, as pointed out previously [27,74], and is also reflected by the present calculations (see Figure 3). It has been pointed out by Leineweber et al. [74] that specific thermodynamic properties of the three phases γ′, α, and ε′ have to be present such that the Gibbs energy of the reaction $\gamma^{\prime }⇌\alpha +\varepsilon^{\prime }$ (e_3_) can change its sign from negative to positive upon increasing temperature. It was proposed that this is caused by different amounts of vibrational entropy acquired by the different phases at *T* > 0 K [74], i.e., already at low temperatures, γ′-Fe_4_N has a higher (e.g., vibrational) heat capacity as compared to α-Fe + ε′-Fe_3_N.

Such a special character of the thermodynamics of γ′-Fe_4_N seems to be confirmed by thermodynamic data predicted in a recent work using first-principles calculations [75]. In that work, vibrational thermodynamic and magnetic properties were worked out for *P* = 0 GPa and *T* > 0 K for α-Fe, the nitrides α″-Fe_16_N_2_ and γ′-Fe_4_N where α″-Fe_16_N_2_ is found stable against α and γ′ at *T* = 0 K. (Note that α″-Fe_16_N_2_ is not considered in the present work but it is, in any case, metastable against α+ε′, as implied by the data listed in Reference [76], although it was apparently not pointed out explicitly. In contrast to γ′-Fe_4_N, there is no experimental evidence that α″-Fe_16_N_2_ gets stable against other nitride phase at any pressure or temperature.) This situation changes with increasing temperature, i.e., the Gibbs energy of the reaction $\alpha^{\prime \prime }⇌\alpha +\gamma$ changes its sign from positive to negative. Comparison of the heat capacity data provided by De Waele et al. [75] indeed imply a higher heat capacity at low temperatures, and thus acquisition of higher entropy with increasing temperature for the γ′ phase as compared to α and α″. The special vibrational behaviour of γ′-Fe_4_N, which would definitely deserve more detailed attention, also explains the stabilisation of γ′ against α + ε′ as encountered experimentally.

The first-principles calculations can also rationalise the increase in the temperature of the reaction $\gamma^{\prime }⇌\alpha +\varepsilon^{\prime }$ (e_3_) with increasing pressure: The larger volume of γ′-Fe_4_N as compared to α + ε′, which is evident from Figure 3b, shows that γ′-Fe_4_N is continuously destabilised with respect to α + ε′-Fe_3_N at high pressure. This means that, although it is entropically stabilised against α + ε′ at low pressure, requiring the occurrence of a reaction $\gamma^{\prime }⇌\alpha +\varepsilon^{\prime }$ (e_3_), γ′-Fe_4_N will be destabilised as the pressure is increased, leading to a shift of the equilibrium temperature of reaction e_3_ to higher temperature. This evolution was already predicted in in our previous work [32] based on experimental crystallographic data and is demonstrated here experimentally (see Section 4.1). According to the DFT calculations and the current experimental data it is presently considered unlikely that γ′ becomes thermodynamically stable at any pressure at *T* ≤ 300 K. Many earlier works have been devoted to studying the RT compression of γ′-Fe_4_N, and it has generally been found that the γ′ phase is retained up to very high pressures. In view of the arguments presented above, the persistence γ′ can only be understood to be the result of metastable retention.

The $\varepsilon_{1}^{\prime }$-, $\varepsilon_{2}^{\prime }$-, and $\varepsilon_{3}^{\prime }$-Fe_4_N model structures all have an energy higher than γ′-Fe_4_N, hence they are also energetically uncompetitive against γ′-Fe_4_N at *T* = 0 K (see Figure 3a and Table 2). The N ordering in $\varepsilon_{1}^{\prime }$ and $\varepsilon_{2}^{\prime }$ much more resembles that of γ′-Fe_4_N, whereas the model structure of $\varepsilon_{3}^{\prime }$-Fe_4_N is derived from ε′-Fe_3_N (see Figure 2 for more information). It is, however, to be noted that the experimentally observed superstructure reflections of ε′ around the Fe_4_N composition are compatible [27] with a superstructure cell which conforms with the unit cell of $\varepsilon_{3}^{\prime }$-Fe_4_N/$\varepsilon^{\prime }$-Fe_3_N and is well established for ε′ nitrides of higher N contents [3,4,5]. This suggests that $\varepsilon_{3}^{\prime }$-derived ordering might be entropically stabilised against $\varepsilon_{1}^{\prime }$ and $\varepsilon_{2}^{\prime }$, in agreement with the relatively easy partial disordering in the N partial structure in ε′ of higher N content [4,5]. Indeed, configurational entropy induced by N disordering may also contribute to the stabilisation of ε′-Fe_4_N against γ′-Fe_4_N, as indicated by the polymorphic reaction $\varepsilon^{\prime }⇌\gamma^{\prime }$ (c_1_) at elevated temperatures (compare Figure 1 and Figure 6a). Additionally, at these high temperatures, ε′ has a lower volume than γ′, as indicated by the negative slope of the univariant line of reaction c_1_ in Figure 8.

All the hcp-based Fe_4_N model structures have a volume smaller than γ′-Fe_4_N (and insignificantly smaller compared to the α + ε′-Fe_3_N mixture). This also implies a destabilisation of γ′-Fe_4_N against any of the considered ε′-Fe_4_N model structures. The energy–volume characteristics of ε′-Fe_4_N as compared to γ′-Fe_4_N have been considered previously [27], and led to the estimation of a theoretical transition pressure of 6 GPa via

(3) $$P=-\frac{u\left({\varepsilon^{\prime }\text{-}\mathrm{Fe}}_{4}N\right)-u\left({\gamma^{\prime }\text{-}\mathrm{Fe}}_{4}N\right)}{V\left({\varepsilon^{\prime }\text{-}\mathrm{Fe}}_{4}N\right)-V\left({\gamma^{\prime }\text{-}\mathrm{Fe}}_{4}N\right)},$$

where u and *V* are the energy and volume determined from DFT calculations. Thereby, temperature effects and instability against α + ε′-Fe_3_N have been neglected. The model structure considered in Reference [27] was described as Fe_24_N_6_ in Reference [3] with a proposed *P*312 symmetry. That structure appears to correspond to the present $\varepsilon_{3}^{\prime }$-Fe_4_N structure, for which compatibility with higher *P*6_3_22 symmetry has been found (see Table 2). For that structure, a transition pressure of 5.7 GPa is estimated in the present work (see Table 2), which agrees well with the value of 6 GPa calculated by Niewa et al. [27]. In agreement, with significantly lower energies than $\varepsilon_{3}^{\prime }$-Fe_4_N, the Fe_4_N model structures $\varepsilon_{1}^{\prime }$ and $\varepsilon_{2}^{\prime }$ yield lower transition pressures of 3.2 GPa and 2.9 GPa, respectively.

The calculated transition pressures suggest the feasibility of a low-temperature pressure-induced polymorphic $\gamma^{\prime }\to \varepsilon^{\prime }$ transition, even if the product phase ε′-Fe_4_N has to be metastable with respect to α + ε′-Fe_3_N. This is in contrast with the apparent high stability of γ′-Fe_4_N at pressures far exceeding 3 GPa. However, the information given in the literature is somewhat conflicting: A partial $\gamma^{\prime }\to \varepsilon^{\prime }$ transformation at RT, starting at *P* > 16 GPa has been reported by References [20,22,60], whereas full retention up to at least 60 GPa has been reported in other works [21,24,25,26]. In view of the different pressure media/experimental setups employed in these studies, it can be concluded in general that non-hydrostatic stress promotes the transition, but full transition can hardly be achieved, especially under quasi-hydrostatic conditions.

Sluggishness of the pressure-induced $\gamma^{\prime }\to \varepsilon^{\prime }$ transformation might be caused by the presence of the nitrogen atoms in the octahedral sites. In contrast to naïve expectation, the change of a fcc to a hcp stacking sequence in Fe_4_N cannot be achieved simply by slip of Shockley partial dislocations, as this would move the interstitial atoms to tetrahedral sites. An appropriate solution to this is so-called synchro-shear [77], where thermally activated jumps of N atoms on appropriate interstitial sites will contribute to the slip and change of the stacking sequence. Such diffusive N–N jumps are, however, expected to be very sluggish at ambient temperature in close-packed cubic and hexagonal iron nitrides [9,78,79]. The involvement of a diffusional-displace mechanism in the $\gamma^{\prime }⇌\varepsilon^{\prime }$ transition is also evident from Figure 4g,h. In summary, it is concluded that the establishment of a true, new equilibrium structure of ε′-Fe_4_N starting from γ′-Fe_4_N is unlikely to be possible merely by increasing pressure at RT.

It is interesting to note that a (partial) structural transition of $\gamma^{\prime }\to \varepsilon^{\prime }$ can be achieved during high-energy ball milling [80]. During ball milling, a large degree of shear deformation is induced in the material. This is accompanied by grain size reduction to the nanoscale and defect formation, both promoting N mobility. Foct et al. [81] have proposed that simultaneous redistribution of N atoms and fcc → hcp transformation of the Fe partial structure facilitate the milling induced transition. Similarly, the $\gamma^{\prime }\to \varepsilon^{\prime }$ transition at *T* ≤ 150 °C is promoted by an increase in atom mobility during Ne ion irradiation [82]. During irradiation, both ballistic jumps and point defect formation promote diffusion [83].

The discussion of phase stability in the Fe–N system at elevated pressure has thus far been confined to pressures relevant for the present study. At pressures typically above 13 GPa at RT, the ε-Fe phase emerges [84]. Due to the α⇌ε transition, the equilibrium α + γ + ε′ (e_4_) has to transfer into a ε + γ + ε′ equilibrium involving at least one more quadruple point and two more univariant equilibria. Due to the steep slope of the α + ε line, the ε + γ + ε′ will potentially emerge within the pressure range of metastable coexistence of α and ε during the pressure-induced α→ε transition. It can be expected that this equilibrium will be present at temperatures much lower than the α + γ + ε triple point of pure Fe such that only the high-temperature equilibria such asd γ + ε′ will be experimentally accessible. Such two-phase equilibrium has been observed at *T* ≥ 400 °C in the pressure range 20–30 GPa by Litasov et al. [23].

In accordance with the data of Litasov et al. [23], it is suggested that the ε′ phase is stable up to at least 30 GPa. At higher pressures, new crystal structures involving N atoms occupying interstitial sites within Fe trigonal prisms can be realised. As is the case for Fe carbides, these structures generally have a markedly lower molar volume than the Fe nitrides reported here. Minobe et al. [25] have shown that at a pressure of 62 GPa a phase transformation of ε′-Fe_7_N_3_ can be induced upon heating. The obtained phase β-Fe_7_N_3_ with space group *P*6_3_*mc* is structurally related to Fe_7_C_3_ and is likely stable at *P* ≥ 43 GPa. Further discussion of Fe nitrides with high N contents, such as NiAs-type FeN [29], FeN_2_ [30] or FeN_4_ [31], and potential phase equilibria involving these phases is beyond the scope of the present work. In any case, the present data show that the γ′ phase is unlikely to occur in the Fe–N system at pressures exceeding 10 GPa, opposing the *P*–*T* projection proposed by Breton et al. [24].

## 5. Conclusions

In the present work, an extensive experimental study of the influence of pressure on phase equilibria in the Fe-rich part of the Fe–N system has been conducted. By imposing pressure in the GPa range to Fe–N phases, decomposition into N depleted Fe and N_2_ gas is effectively avoided, which allows the study of equilibria and stability of the condensed phases in the system in a wide temperature range. The present results imply that the γ′ phase is thermodynamically stable within a confined *P*–*T* window, both with respect to decomposition into Fe + N_2_ and with respect to transformation into other Fe–N phases. A *P*–*T* projection of the univariant phase equilibria in the Fe–N system has been constructed, which is consistent with the experimentally observed phase relations. Due to its high molar volume, the temperature range of stability of γ′-Fe_4_N_1−*x*_ constricts with increasing pressure, resulting in its disappearance around $5.0_{-0.5}^{+1.1}\;\mathrm{GPa}$ and $375_{-25}^{+56}$ °C. After its disappearance, the terminal solid solution phases of α-Fe, γ-Fe (and ε-Fe) and the ε′-Fe_3_N_1+*x*_ nitride remain the only stable phases in the system up to a composition of at least Fe_3_N.

Based on DFT calculations, existing literature data, and present experimental results, phase stability in the Fe–N system has been discussed and can be summarised as follows: The γ′ phase is unstable at *T* = 0 K with respect to decomposition into α-Fe + ε′-Fe_3_N at any pressure. On the other hand, γ′-Fe_4_N is more stable than different model structures of hcp-based ε′-Fe_4_N at 0 K. The lower molar volume of ε′-Fe_4_N, however, suggests the feasibility of a pressure-induced polymorphic reaction to (metastable) ε′-Fe_4_N. The apparent stability of γ′-Fe_4_N with respect to such transition is interpreted as metastable retention caused by a kinetic barrier due to low N mobility during a synchro-shear transformation mechanism.

At elevated temperatures, γ′-Fe_4_N is entropically stabilised against α + ε′-Fe_3_N, resulting in the emergence of the eutectoid $\gamma^{\prime }⇌\alpha +\varepsilon^{\prime }$. In the present study, a direct decomposition $\gamma^{\prime }\to \alpha +\varepsilon^{\prime }$ has been observed for the first time. It is suggested that vibrational contributions to entropy cause the stabilisation of γ′-Fe_4_N at intermediate temperatures. At high temperatures, however, a congruent transformation $\gamma^{\prime }⇌\varepsilon^{\prime }$ is experimentally observed. This means that the entropy contribution to the Gibbs energy of ε′-Fe_4_N, which is less stable than γ′-Fe_4_N at 0 K, needs to outgrow that of γ′-Fe_4_N. In contrast to γ′-Fe_4_N, ε′-Fe_4_N has more structural degrees of freedom in terms of N ordering/partial disordering. Therefore, it is suggested that configurational entropy may play a crucial role in the stabilisation of ε′-Fe_4_N at elevated temperatures. At high pressure, the volume-dependent energy terms outweigh the entropy terms, and the γ′ phase becomes thermodynamically unstable at any temperature due to its high molar volume.

## Figures and Tables

**Table 1:** Relevant phases studied in the present paper and their crystallographic models considered in the course of phase identification and lattice parameter determination. For the models used in the DFT calculations, see Table 2.

Phase Identifier	Fe Basis Structure	Space Group	Fe Atoms per Employed Unit Cell	Lattice Parameters of the Employed Unit Cell	Reference for Structure Model
α/α-Fe(N)	bcc	*Im* $\bar{3}$ *m*	2	*a*_α_ = *a*_bcc_	[6]
γ/γ-Fe(N)	fcc	*Fm* $\bar{3}$ *m*	4	*a*_γ_ = *a*_fcc_	[6]
γ′/γ′-Fe_4_N	fcc	*Pm* $\bar{3}$ *m*	4	*a*_γ__′_ = *a*_fcc_	[2]
ε′/ε′-Fe_3_N_1+*x*_ ^a^	hcp	*P*6_3_22	6	*a*_ε__′_ = 3^1/2^ *a*_hcp_, *c*_ε__′_ = *c*_hcp_	[4,5]

^a^ Using the prime notation to indicate the state of order, the differently ordered hcp-based ζ-nitride (ζ-Fe_2_N; not regarded as relevant for this work) might be referred to as ε″. For the sake of brevity, we will refer to the ε′ nitride of composition Fe_3_N_0.75_ as ε′-Fe_4_N.

**Table 2:** Model structures for and results of the DFT calculations including crystallographic information on the employed model structures of relevant Fe–N phases with different N contents. Given are relaxed lattice parameters and relationship to unit cells of the bcc, fcc, and hcp Fe allotropes, energy, and volume differences per atom (Fe + N) with respect to a phase mixture α-Fe + ε′-Fe_3_N and equilibrium pressures *P*_0_ for a potential $\gamma^{\prime }\to \varepsilon^{\prime }$ transition at 0 K, estimated according to Equation (3).

Model Structure for DFT	Space Group	Fe/N Atoms per Unit Cell	Lattice Parameters	Δ*u* ^a^ [meV]	Δ*V* ^a^ [Å^3^]	*µ*^b^ [μ_B_]
α-Fe	*Im* $\bar{3}$ *m*	2/0	*a*_α_ = 2.8315 Å = *a*_bcc_	0	0	2.20
γ′-Fe_4_N	*Pm* $\bar{3}$ *m*	4/1	*a*_γ_ = 3.7877 Å = *a*_fcc_	+12.5	+0.53	2.48
$\varepsilon_{1}^{\prime }$-Fe_4_N	*P*6_1_22	24/6	$a_{\varepsilon_{1}^{\prime }}$ = 5.2541 Å = 2 *a*_hcp_,	+21.9	−0.03	1.97
			$c_{\varepsilon_{1}^{\prime }}=$ 12.8796 Å = 3*c*_hcp_			
$\varepsilon_{2}^{\prime }$-Fe_4_N	F2dd	32/8	$a_{\varepsilon_{2}^{\prime }}$ = 5.2705 Å ≈ 2*a*_hcp_,	+20.0	−0.00	1.99
			$b_{\varepsilon_{2}^{\prime }}$ = 9.1055 ≈ 2 × 3^1/2^*a*_hcp_,			
			$c_{\varepsilon_{2}^{\prime }}$ = 8.61456 = 2*c*_hcp_			
$\varepsilon_{3}^{\prime }$-Fe_4_N	*P*6_3_22	24/6	$a_{\varepsilon_{3}^{\prime }}$ = 2 × 3^1/2^*a*_hcp_ = 9.1409 Å,	+34.3	−0.00	2.02
			$c_{\varepsilon_{3}^{\prime }}$ = 4.2839 Å = *c*_hcp_			
ε′-Fe_3_N	*P*6_3_22	6/2	*a*_ε__′_ = 4.6475 Å = 3^1/2^*a*_hcp_,	0	0	2.04
			*c*_ε__′_ = 4.3119 Å = *c*_hcp_			

^a^ Energy/volume of formation from α + ε′-Fe_3_N corresponding to the vertical distance of the energy value from the line connecting the energy values of α + ε′-Fe_3_N in Figure 3. Note that in Figure 3 energy of formation values from α-Fe + N_2_ are shown. ^b^ Average magnetic moment per Fe atom.

**Table 3:** Conditions of the high-pressure heat treatments—estimated sample pressure *P*, homogenisation temperature *T*_1_ and time *t*_1_, temperature of the final annealing step *T*_2_ and annealing time *t*_2_, and initial phase composition and phases present after high-pressure heat treatment.

Sample ID	*P* [GPa]	*T*_1_ [°C]	*t*_1_ [h]	*T*_2_ [°C]	*t*_2_ [h]	Initial Phases	Product Phases
2-400	2	900	1	400	4	α + γ′	α + γ′
2-500	2	–	–	500	4	α + γ′	γ + γ′
2-550a	2	–	–	550	4	α + γ′	γ + ε′
2-550b	2	–	–	550	4	γ′	γ′ + ε′
2-600	2	–	–	600	4	γ′	ε′
2-800	2	–	–	800	1	α + γ′	γ + ε′
2-1000	2	–	–	1000	0.25	α + γ′	ε′
3-400	3	–	–	400	1	γ′	γ′ + ε′
4-250	4	–	–	250	4	γ′	γ′ + (ε′)
4-300	4	–	–	300	4	γ′	γ′ + ε′
4-400a	4	1000	1	400	4	α + γ′	α + γ′
4-400b	4	–	–	400	4	γ′	γ′ + ε′
4-450	4	1000	0.5	450	4	α + γ′	γ + γ′ + ε′
4-500	4	–	–	500	1	γ′	ε′
4-600	4	–	–	600	1	α + γ′	γ + ε′
4-1000	4	–	–	1000	1	α + γ′	ε′
4.5-400	4.5	1000	0.5	400	4	α + γ′	α + γ + γ′ + ε′
5-300	5	–	–	300	4	γ′	α + γ′ + ε′
5-350	5	–	–	350	4	γ′	α + γ + γ′ + ε′
5-400	5	–	–	400	4	γ′	γ + ε′
6-400	6	–	–	400	4	γ′	γ + ε′
8-400	8	–	–	400	4	γ′	γ + ε′

**Table 4:** Primary data obtained from XRD analysis—weight fractions *w* of the phases, lattice parameters *a* and *c*, and volumes per Fe atom *V*_Fe_ are given for the α and γ solid solution phases as well as the nitride phases γ′ and ε′.

ID	α			γ			γ′			ε′			
	*w*	*a*	*V*_Fe_	*w*	*a*	*V*_Fe_	*w*	*a*	*V*_Fe_	*w*	*a*	*c*	*V*_Fe_
	[wt%]	[Å]	[Å³]	[wt%]	[Å]	[Å³]	[wt%]	[Å]	[Å³]	[wt%]	[Å]	[Å]	[Å³]
2-400	30.1	2.8687	11.80				69.9	3.7968	13.68				
2-500				40.4	3.6443	12.10	59.6	3.7912	13.62				
2-550a				34.4	3.6521	12.18				65.6	4.5912	4.3342	13.19
2-550b							18.8	3.7920	13.63	81.2	4.6283	4.3555	13.47
2-600										100	4.6271	4.3554	13.46
2-800				8.9	3.6542	12.20				91.1	4.5573	4.3019	12.90
2-1000										100	4.5500	4.2959	12.84
3-400							84.0	3.7967	13.68	16.0	4.6685	4.3715	13.75
4-250 ^a^							100	3.7999	13.72				
4-300							93.6	3.7984	13.70	6.4	4.6832	4.3802	13.87
4-400a	26.3	2.8688	11.81				73.7	3.7948	13.66				
4-400b							79.3	3.7957	13.67	20.7	4.6504	4.3654	13.63
4-450				37.8	3.6459	12.12	30.0	3.7916	13.63	32.2	4.6056	4.3456	13.30
4-500										100.0	4.6254	4.3546	13.45
4-600				28.6	3.6527	12.18				71.4	4.5814	4.3274	13.11
4-1000										100.0	4.5492	4.2949	12.83
4.5-400	23.2	2.8679	11.79	8.0	3.6474	12.13	44.7	3.7941	13.65	24.1	4.6430	4.3595	13.56
5-300	7.1	2.8692	11.81				55.1	3.7967	13.68	37.8	4.6724	4.3763	13.79
5-350	5.9	2.8686	11.80	3.0	3.6456	12.11	22.8	3.7946	13.66	68.3	4.6503	4.3661	13.63
5-400				10.6	3.6470	12.13				89.4	4.6420	4.3627	13.57
6-400				15.0	3.6468	12.13				85.0	4.6475	4.3658	13.61
8-400				21.4	3.6440	12.10				78.6	4.6591	4.3706	13.69

^a^ Only one faint reflection of the ε′ phase is visible and was therefore neglected in refinement.

**Table 5:** Phase constitution data: N contents in terms of molar fractions *x*_N_ determined from the unit cell volumes of the phases indicated above (see text for details), N contents *x*_N_ of the ε′ phase calculated by means of Equation (2), N contents *x*_N,LR_ calculated by means of the lever rule in Equation (1) and total average N contents of the samples calculated from N contents and phase fractions.

Sample ID	α	γ	γ′	ε′		Average
	*x*_N_ [at%]	*x*_N_ [at%]	*x*_N_ [at%]	*x*_N_ [at%]	*x*_N,LR_ [at%]	*x*_N_ [at%]
2-400	0.3		19.9			14.7
2-500		9.2	19.5			15.5
2-550a		10.1		18.0	17.7	15.4
2-550b			19.5	20.5		20.4
2-600				20.5	20.2	20.5
2-800		10.3		15.4	15.6	14.9
2-1000				14.8	15.2	14.8
3-400			19.9	23.2		20.4
4-250			20.1			20.1
4-300			20.0	24.3		20.3
4-400a	0.3		19.7			15.2
4-400b			19.8	22.0		20.3
4-450		9.4	19.5	19.1		15.7
4-500				20.4	20.2	20.4
4-600		10.2		17.3	17.1	15.3
4-1000				14.8	15.2	14.8
4.5-400	0.2	9.5	19.7	21.4		15.4
5-300	0.4		19.9	23.6		20.2
5-350	0.3	9.3	19.7	22.0		20.1
5-400		9.5		21.5	21.3	20.3
6-400		9.5		21.9	21.9	20.2
8-400		9.1		22.6	22.9	20.0

## Data Availability

Data is contained within the article or Supplementary Materials or otherwise available upon reasonable request from the corresponding author A.L.

## Supplementary Materials

The following are available online at https://www.mdpi.com/article/10.3390/ma14143963/s1.
