# Supplementary material for: Phase Stability of Iron Nitride Fe4N at High Pressure—Pressure-Dependent Evolution of Phase Equilibria in the Fe–N System
Source: Materials (Basel). 2021 Jul 15;14(14):3963. doi: 10.3390/ma14143963 (PMC8307547; doi:10.3390/ma14143963)
Supplement: Supplementary file 1 [file materials-14-03963-s001.zip › suppl.pdf]

Supplementary Material

1. *Experimental setup*

Figure S1 shows schematic sketches of the pressure cells employed in the present study. The sketches are drawn to scale, for more details on the exact measurements see text of the original manuscript.

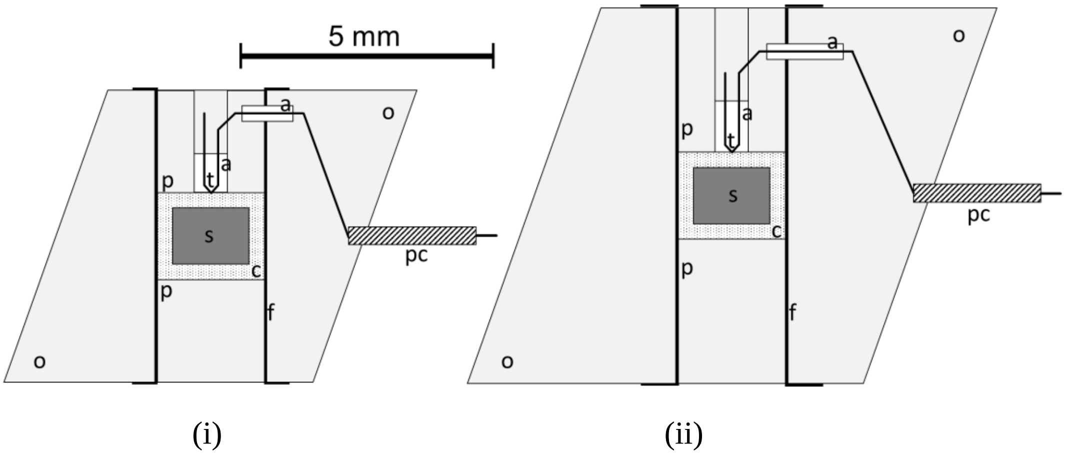

Figure S1. Schematic drawing of the octahedral pressure cells: (i) 14 mm and (ii) 18 mm octahedral edge length. The pressure cells are composed of the following components: s - sample, c - CsCl capsule, p - m-ZrO_2 plugs, t - W/Re type C thermocouple junction, a - Al_2O_3 capillary, f - metal foil furnace (= resistive heater), o - m-ZrO_2 octahedral pressure medium, pc - Cu protective coils.

2. *Pressure calibration for multi-anvil experiments*

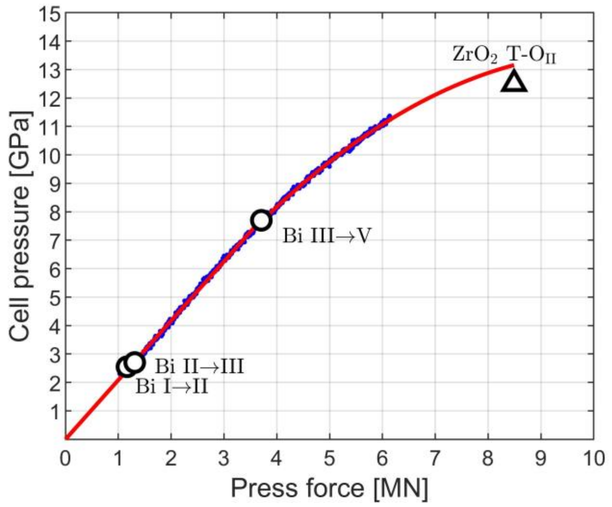

Figure S2. Pressure calibration curve of the 14/8 assembly with m-ZrO_2 pressure medium. Circles indicate phase transitions of Bi which have been used for pressure calibration. The triangle in (i) indicates conditions of occasional orthorhombic-II-ZrO_2 formation.

Figure S2 shows the calibration curve of the 14/8 assembly used in most of the experiments in this study. The curve has been used to estimate the sample pressure within the centre of the pressure cell from the press force of the uniaxial press. The calibration data have been determined in an external room temperature calibration experiment without sample by means of four-probe measurements of resistance changes of Bi during phase transformations (see Ref. [46] for more details). The Bi I→II, II→III and III→V transitions pressures were adopted as 2.55 GPa [43], 2.70 GPa [44] and 7.7 GPa [43], respectively. An exemplary dataset of the resistance changes in the 14/8 calibration experiment is shown in Figure S3. The continuous resistance change of a second calibrant, consisting of a small coil of Manganin® wire (Isabellenhütte Heusler GmbH) cast in epoxy resin, has been measured simultaneously and was used to extrapolate the pressure calibration beyond the pressure of the

last Bi transition up to the maximum applied press load of 8.5 MN. In some of our experiments, typically at $T \geq 900$ °C small amounts of the quenchable orthorhombic-II modification of ZrO_2 were formed in the high temperature zone around the furnace. This result indicates that the sample was subject to a pressure of at least 12.5 GPa [45] which agrees well with the pressure of 13 GPa predicted by the calibration curve.

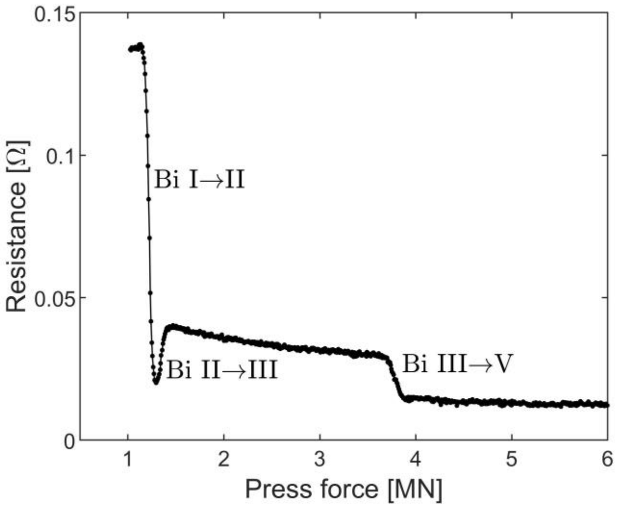

Figure S3. Resistance changes of a Bi calibrant during a 14/8 calibration experiment.

For the 18/12 type assembly a calibration experiment similar to that described for the 14/8 assembly has been conducted. Pronounced resistance changes, typical for the Bi I→II and II→III transitions, have been observed between 1.93 – 2.12 and 2.12 – 2.31 MN press force, respectively. According to this, a press force of 1.61 MN was used in order to conduct heat treatments at sample pressure of 2 GPa.

3. *X-ray diffraction data*

3.1. Starting materials

Figure S4 shows the XRD patterns of the γ'-Fe_4N initial powders and γ'-Fe_4N+α-Fe powder mixture used in the present work. The grey lines show the difference between measured and calculated intensities, which are shown in open black circles and coloured lines respectively. Indices are shown above the corresponding reflections. Superstructure reflections due to N ordering in the γ' nitride are marked with an asterisk.

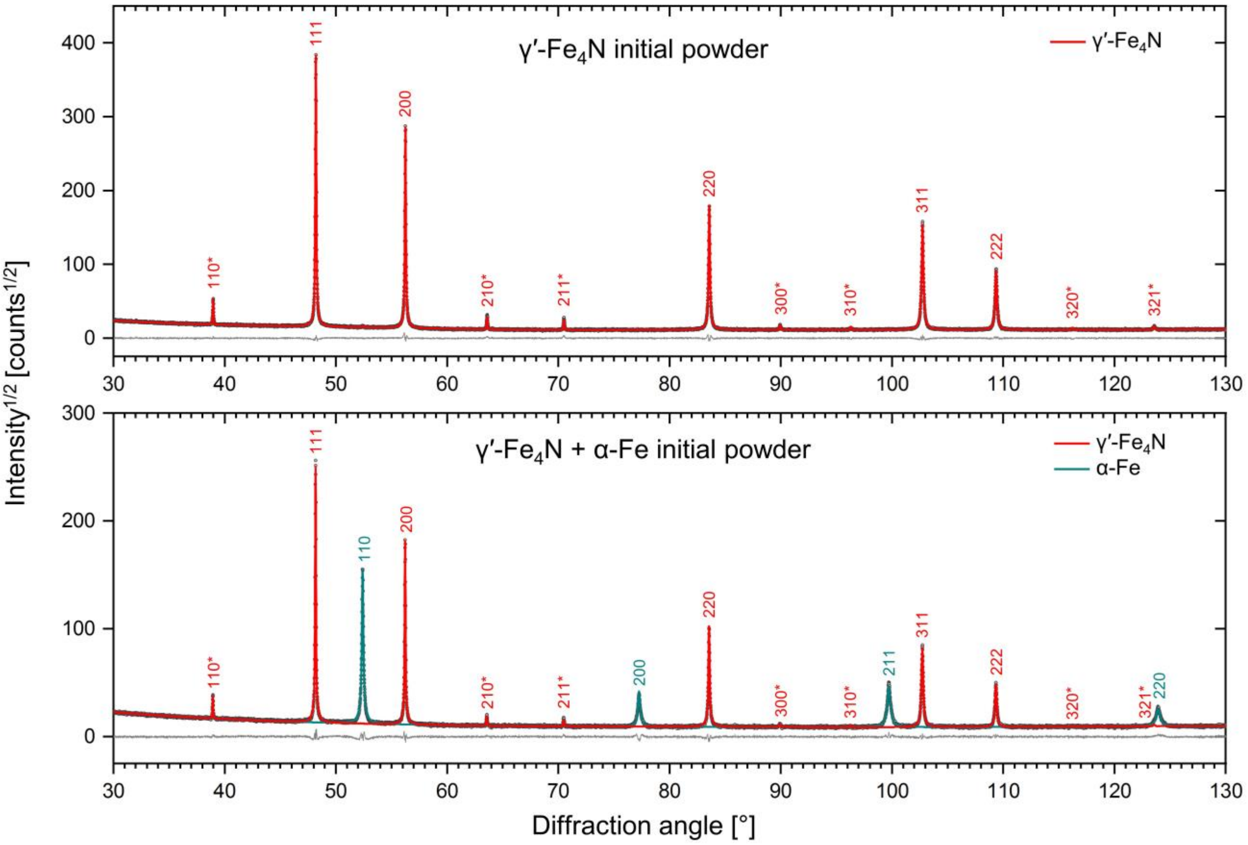

Figure S4. XRD patterns of the two initial powders used in the present study. For details see text.

3.2. Samples heat treated at high pressure

Figures S5–S11 show the diffraction patterns of the samples heat treated at high pressure. The patterns are labelled with the sample names, for example 4-400, meaning the sample has been heat treated at 4 GPa and 400 °C. Indices are shown above the corresponding reflections in colour of the respective phase. Only discernible reflections are indexed and respective superstructure reflections are marked with an asterisk. Measured intensities, calculated intensities are plotted in grey circles, coloured lines respectively. For most samples the calculated intensities are the result of Rietveld refinement. Only single-phase samples 2-1000 (Figure S6) and 4-1000 (Figure S8) were treated with a Pawley fit due to unusual intensity ratios of the reflections, which result from texture and bad crystallite statistics. The difference curves are shown, sometimes with arbitrary constant offsets, as solid grey lines below the diffraction patterns.

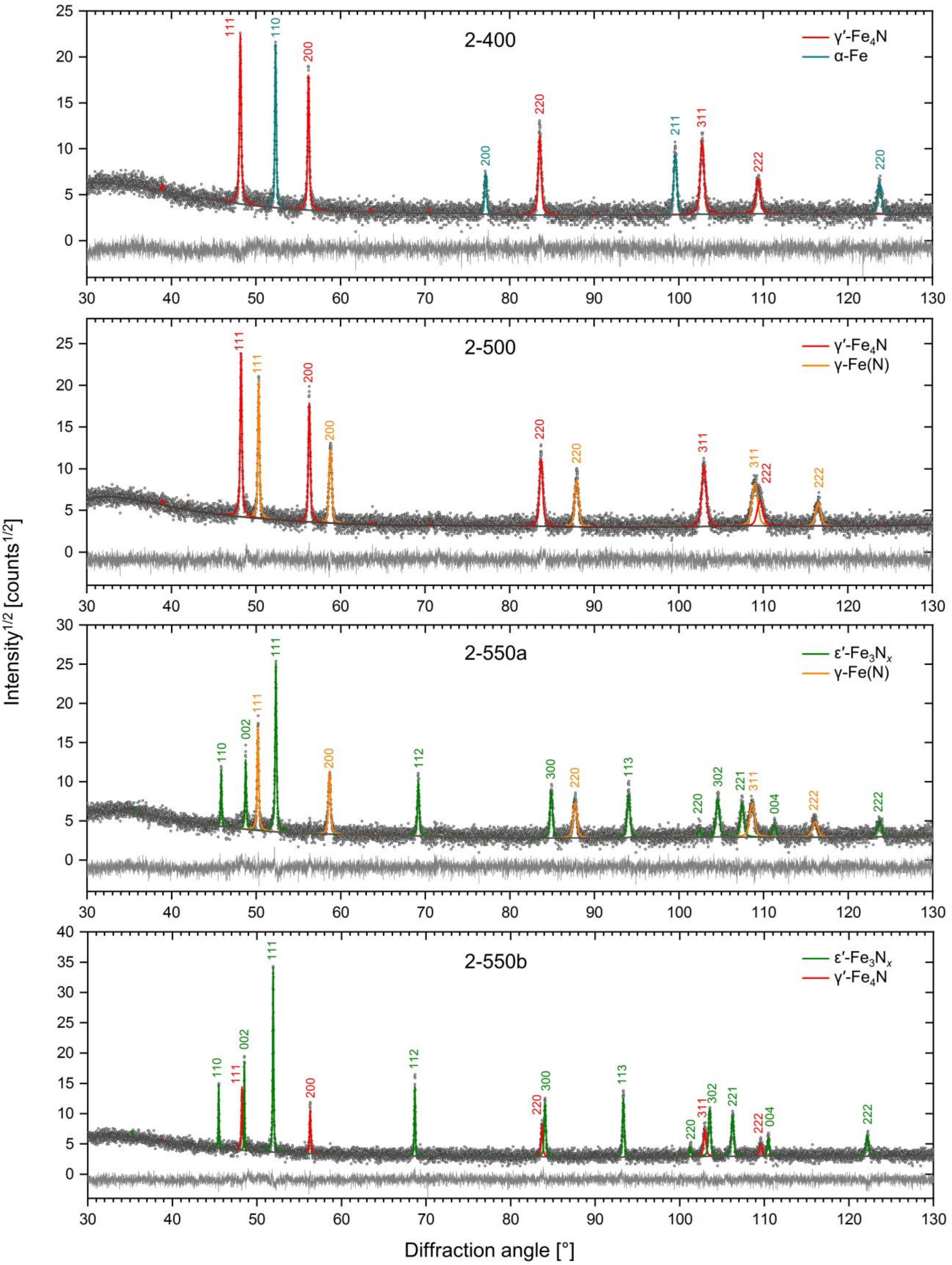

Figure S5. XRD patterns of samples heat treated at 2 GPa and different temperatures. For details see text.

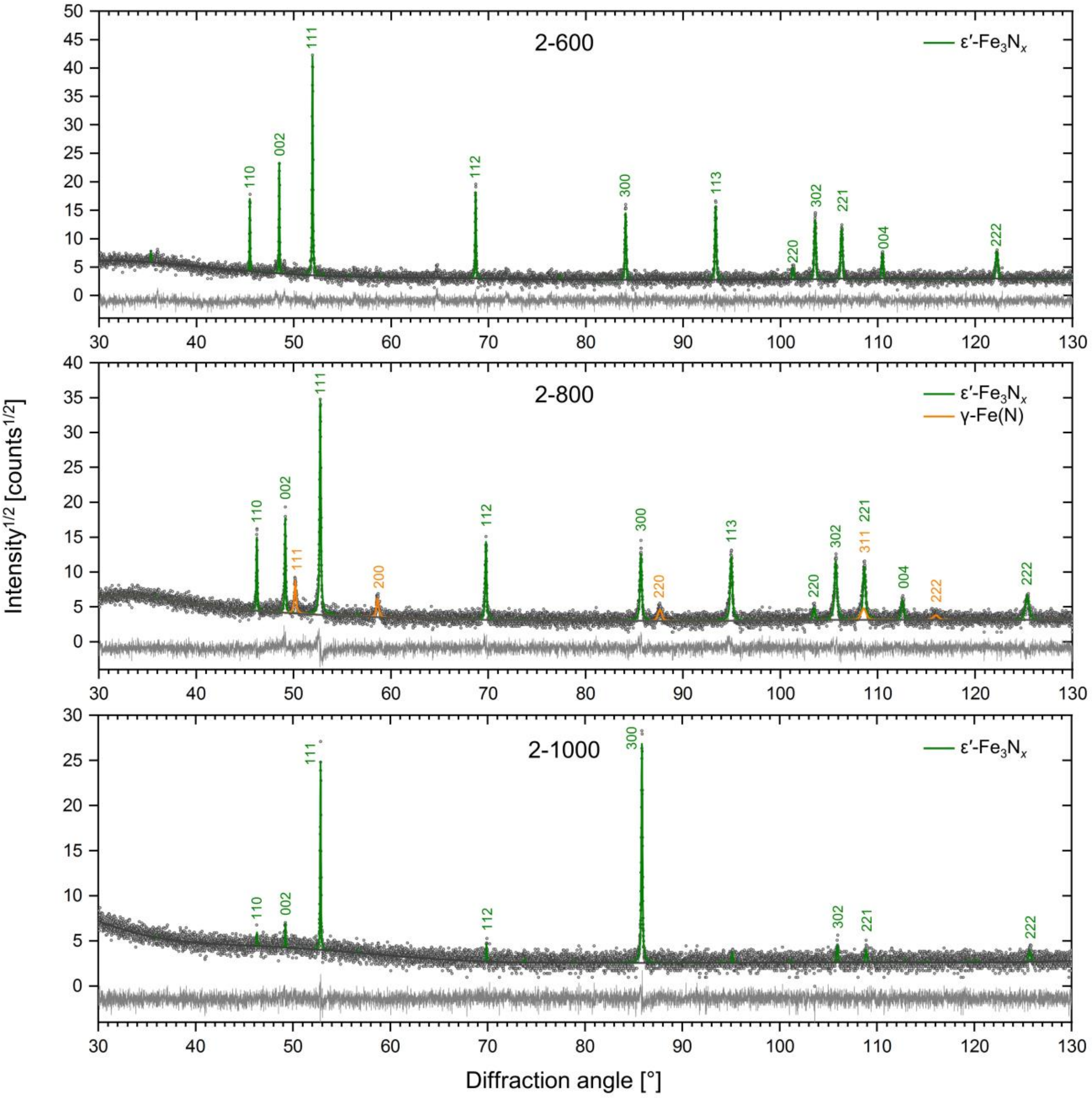

Figure S6. XRD patterns of samples heat treated at 2 GPa and different temperatures. For details see text.

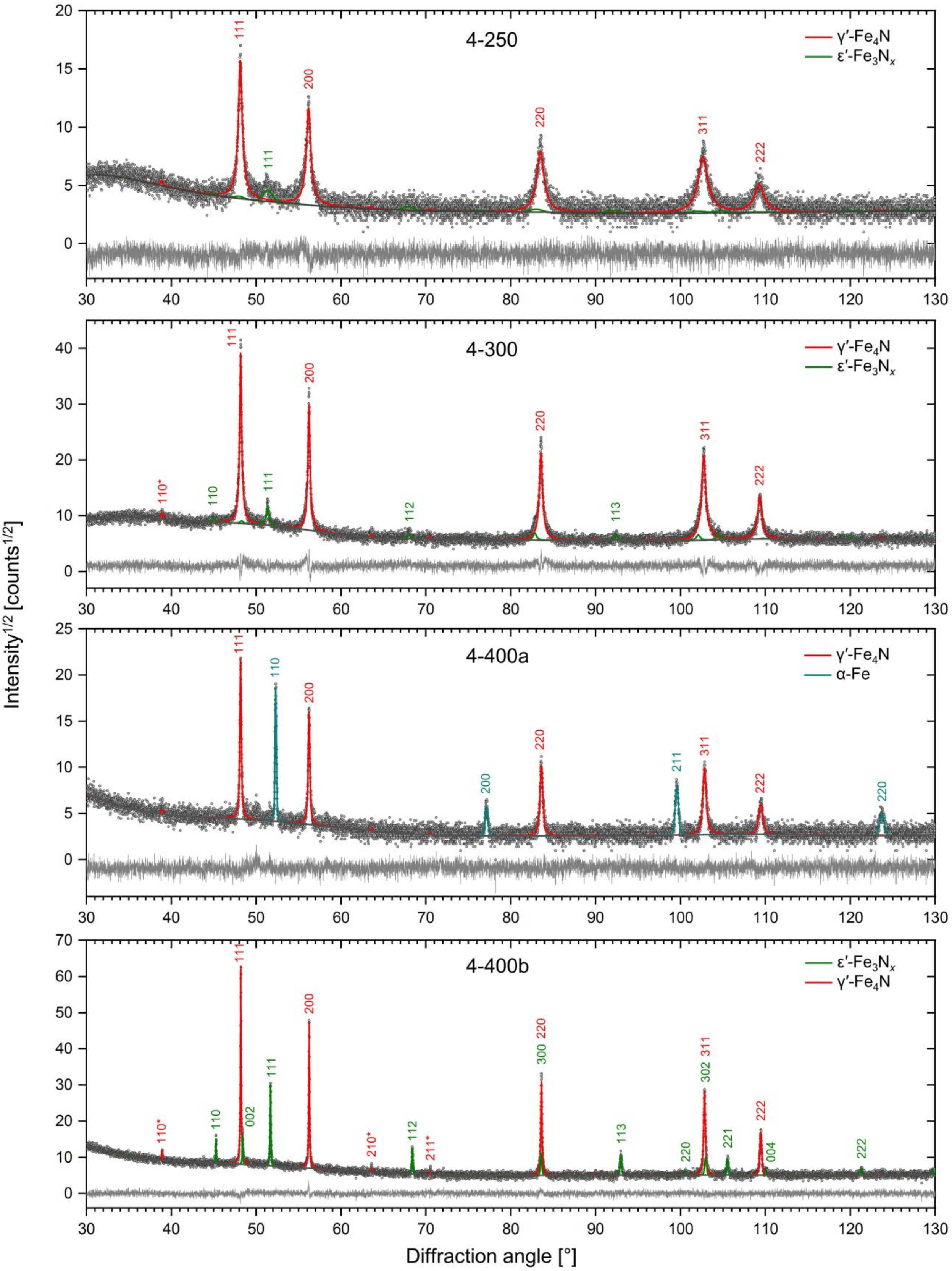

Figure S7. XRD patterns of samples heat treated at 4 GPa and different temperatures. For details see text.

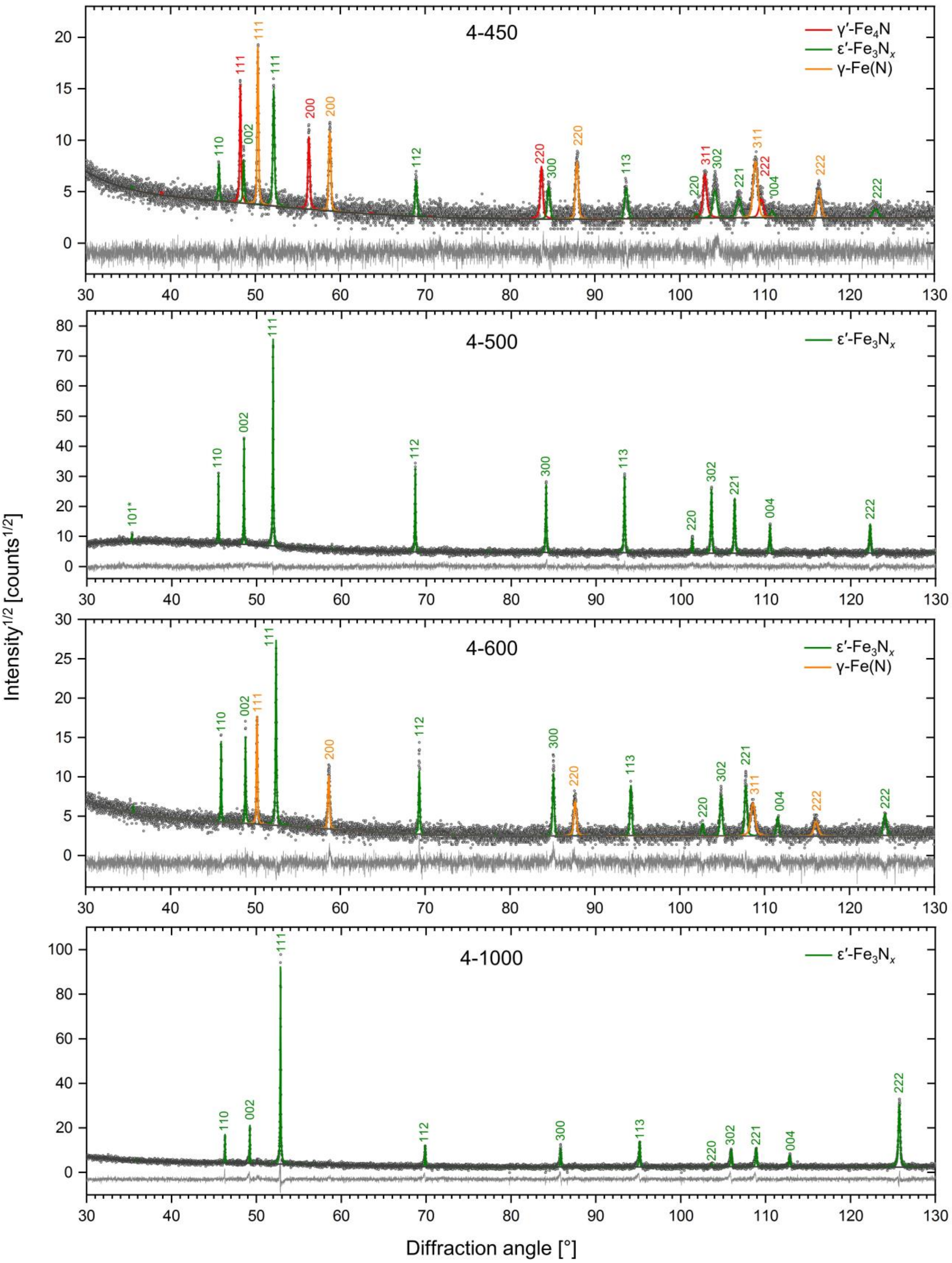

Figure S8. XRD patterns of samples heat treated at 4 GPa and different temperatures. For details see text.

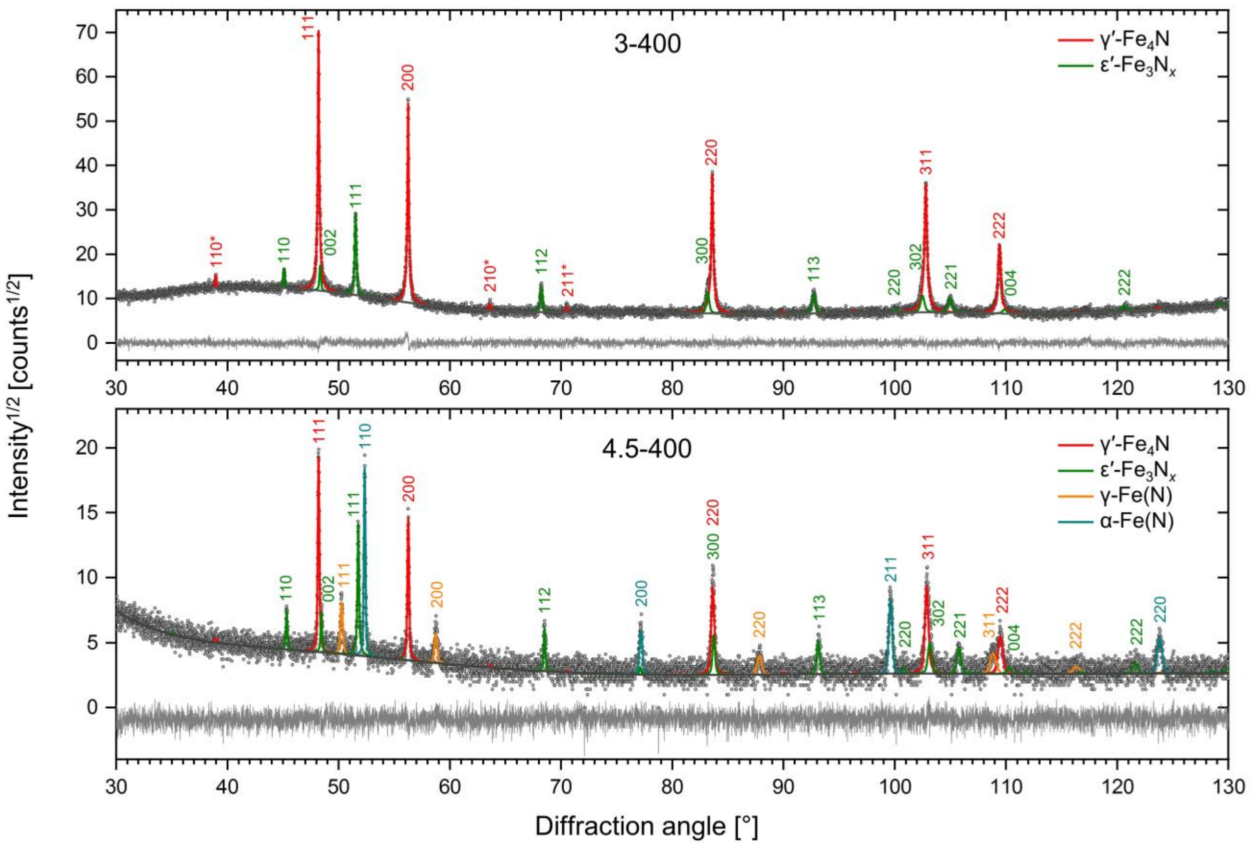

Figure S9. XRD patterns of samples heat treated at 400°C and different pressures. For details see text.

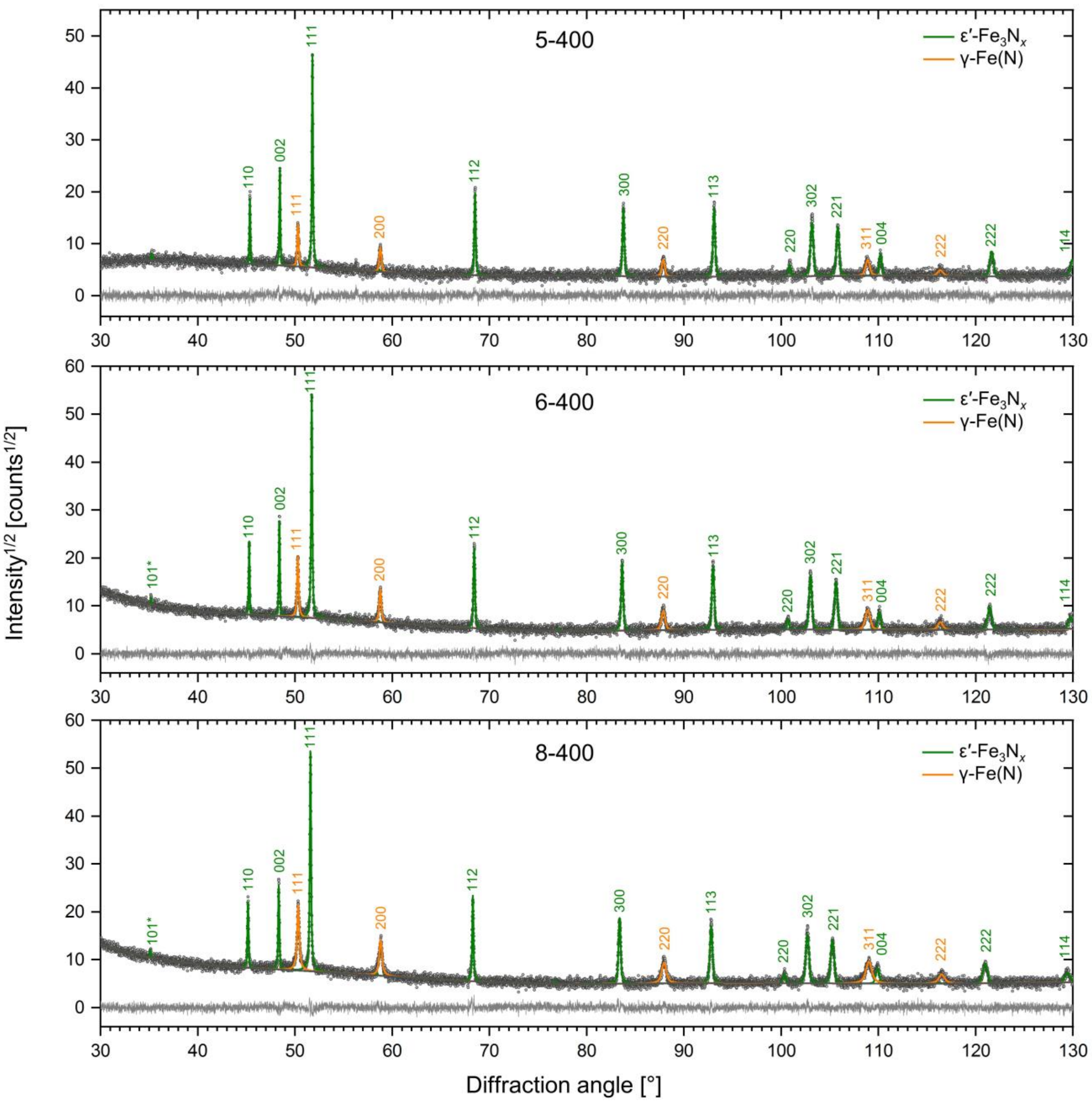

Figure S10. XRD patterns of samples heat treated at 400°C and different pressures. For details see text.

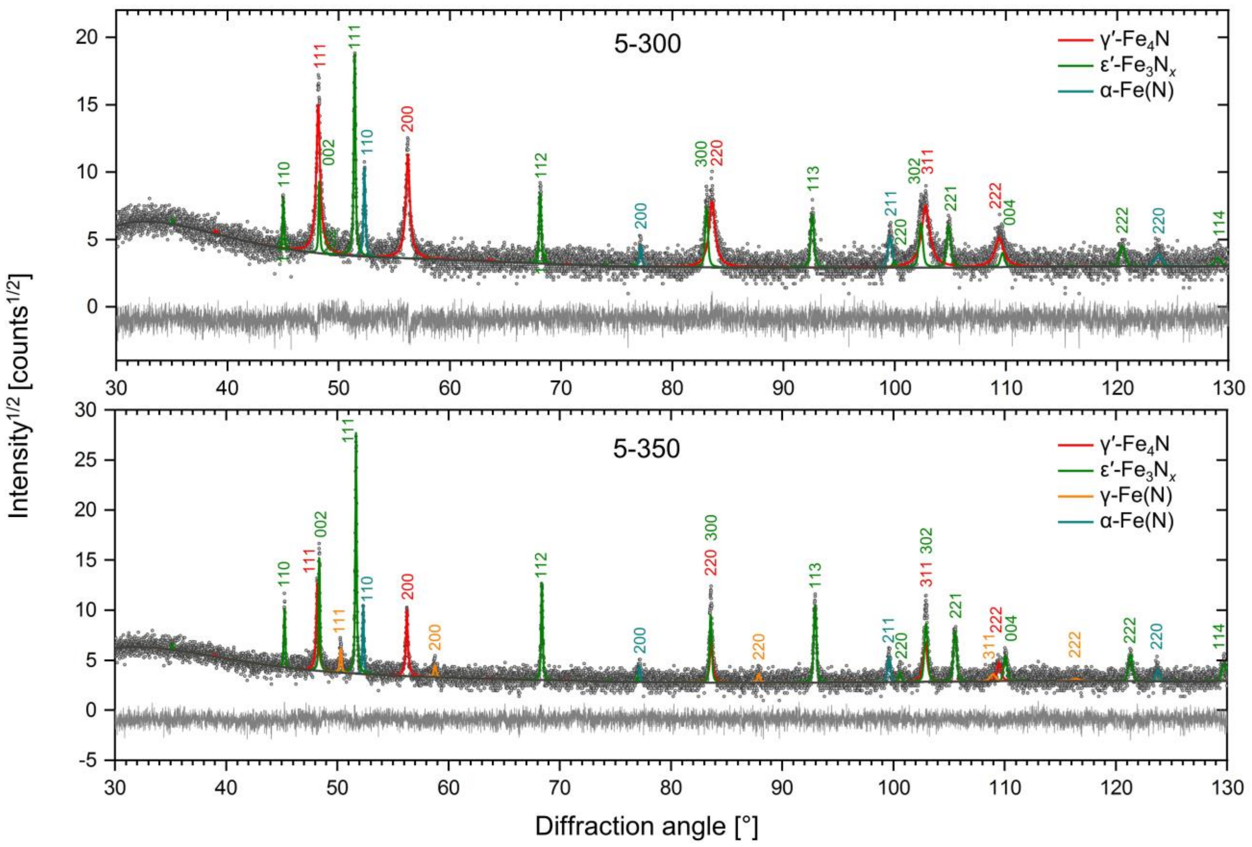

Figure S11. XRD patterns of samples heat treated at 5 GPa and different temperatures. For details see text.

References

43. Rubie, D.C. Characterising the sample environment in multianvil high-pressure experiments. *Phase Transitions* **1999**, *68*, 431–451, doi:10.1080/01411599908224526.

44. Bundy, F.P. Phase Diagram of Bismuth to 130 000 kg/cm², 500°C. *Phys. Rev.* **1958**, *110*, 314–318, doi:10.1103/PhysRev.110.314.

45. Ohtaka, O.; Fukui, H.; Kunisada, T.; Fujisawa, T.; Funakoshi, K.; Utsumi, W.; Irifune, T.; Kuroda, K.; Kikegawa, T. Phase relations and equations of state of ZrO_2 under high temperature and high pressure. *Phys. Rev. B - Condens. Matter Mater. Phys.* **2001**, *63*, 1741081–1741088, doi:10.1103/PhysRevB.63.174108.

46. Schwarz, M.R. Multianvil calibration and education: A four probe method to measure the entire force-versus-pressure curve in a single run - Performed as an interdisciplinary lab-course for students. *J. Phys. Conf. Ser.* **2010**, *215*, doi:10.1088/1742-6596/215/1/012193.
